# Alterations in microbiota of patients with COVID‐19: implications for therapeutic interventions

**DOI:** 10.1002/mco2.513

**Published:** 2024-03-15

**Authors:** Yong Qiu, Chunheng Mo, Lu Chen, Wanlin Ye, Guo Chen, Tao Zhu

**Affiliations:** ^1^ Department of Anesthesiology National Clinical Research Center for Geriatrics and The Research Units of West China (2018RU012) West China Hospital Sichuan University Chengdu China; ^2^ Laboratory of Anesthesia and Critical Care Medicine National‐Local Joint Engineering Research Center of Translational Medicine of Anesthesiology West China Hospital Sichuan University Chengdu China; ^3^ Key Laboratory of Birth Defects and Related Diseases of Women and Children of MOE State Key Laboratory of Biotherapy West China Second University Hospital Sichuan University Chengdu China

**Keywords:** COVID‐19, gut microbiota, immunity, probiotics, vaccine

## Abstract

Severe acute respiratory syndrome coronavirus 2 (SARS‐CoV‐2) recently caused a global pandemic, resulting in more than 702 million people being infected and over 6.9 million deaths. Patients with coronavirus disease (COVID‐19) may suffer from diarrhea, sleep disorders, depression, and even cognitive impairment, which is associated with long COVID during recovery. However, there remains no consensus on effective treatment methods. Studies have found that patients with COVID‐19 have alterations in microbiota and their metabolites, particularly in the gut, which may be involved in the regulation of immune responses. Consumption of probiotics may alleviate the discomfort caused by inflammation and oxidative stress. However, the pathophysiological process underlying the alleviation of COVID‐19‐related symptoms and complications by targeting the microbiota remains unclear. In the current study, we summarize the latest research and evidence on the COVID‐19 pandemic, together with symptoms of SARS‐CoV‐2 and vaccine use, with a focus on the relationship between microbiota alterations and COVID‐19‐related symptoms and vaccine use. This work provides evidence that probiotic‐based interventions may improve COVID‐19 symptoms by regulating gut microbiota and systemic immunity. Probiotics may also be used as adjuvants to improve vaccine efficacy.

## Graphical abstract

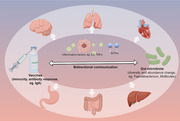

## 1 INTRODUCTION

During the coronavirus disease (COVID‐19) pandemic, more than 702 million people were infected with severe acute respiratory syndrome coronavirus 2 (SARS‐CoV‐2) and over 6.9 million died, while new variants continue to emerge. Patients with COVID‐19 may suffer from fever, sore throat, cough, diarrhea, sleep disorders, anxiety, depression, and cognitive impairment, which is associated with long COVID symptoms during recovery.^1^, ^2^, ^3^, ^4^ Despite some clinical trials of drugs to improve COVID‐19 symptoms, there remains no consensus on effective treatment methods, and immunocompromised elderly people and children need special attention.^5^, ^6^, ^7^, ^8^

Previous studies have suggested that patients with COVID‐19 in the acute or recovery phase exhibit changes in the microbiota, especially the gut microbiota. The alterations in these microorganisms and their metabolites may be involved in the regulation of inflammation and the immune response to mediate virus progression and symptoms. However, the pathophysiological process underlying the alleviation of COVID‐19‐related symptoms and complications by targeting the microbiota remains unclear.^9^, ^10^, ^11^, ^12^ Here, we summarize the latest research and evidence on the COVID‐19 pandemic, as well as the symptoms and vaccine use, with a focus on the relationship between microbiota alterations and COVID‐19‐related symptoms and vaccine use. We also examined the potential value of probiotics as adjuvants in vaccines and in alleviating COVID‐19 symptoms. Thus, this study provides ideas for targeted and individualized intervention for treating COVID‐19 and related complications.

In this review, we systematically explored the symptoms of COVID‐19, the available vaccines, dynamic changes in microbiota, and microbiota‐based implications for therapeutic interventions. We also summarize and discuss the underlying mechanisms, changes in microbiota during acute infection and recovery, gut microbiota dysbiosis and symptoms, vaccine‐associated microbiota changes, probiotic‐related treatment, and adjuvants that improve vaccine potency.

## 2 PANDEMIC, SYMPTOMS, AND VACCINES OF THE COVID‐19

### 2.1 Prevalence and direction

Although the WHO declared the end of the pandemic, biologically relevant genome sequencing analyses of SARS‐CoV‐2 have indicated continued viral variability. The evolution of and change in virus lineages have underlying genetic mechanisms, with evolved strains showing stronger infectivity and immune escape ability. Studies on the affinity between the spike protein of different SARS‐CoV‐2 variants and the angiotensin‐converting enzyme 2 (ACE2) receptor on the membrane of respiratory system cells have revealed that the virus is constantly mutating, resulting in immune escape. Viruses and host immunity may also be correlated, indicating a certain evolutionary direction.^13^, ^14^, ^15^, ^16^ It has been reported that approximately 10% of hospitalized patients are susceptible to COVID‐19, which is associated with prolonged hospital stay and increased risk of postoperative complications, particularly in elderly patients with diabetes, chronic obstructive pulmonary disease, or other frail or immune abnormalities. The increase in COVID‐19 cases will bring additional challenges and increase global medical burden. Furthermore, the potential harm and long‐term impact of COVID‐19 on children's health, psychology, and development deserves more attention, especially when adolescent mental health problems are prone to occur, as is becoming increasingly common. Therefore, it is necessary to explore new effective strategies for early intervention or prevention.^17^, ^18^, ^19^, ^20^, ^21^

### 2.2 Symptoms and the long COVID

#### 2.2.1 Lingering symptoms during recovery

The symptoms of COVID‐19 are heterogeneous, with those of acute infection including include dyspnea, diarrhea, sleep disorders, and olfactory abnormalities, which are often more serious in female patients. Additionally, the incidence of muscle pain and headache can reach 75% in the short term. Patients may also suffer from insomnia, anxiety, and persistent gastrointestinal symptoms. A previous study demonstrated that at 6 months after infection, the incidence of fatigue or muscle weakness was approximately 52%, while that of sleep disorders and anxiety or depression was more than 20%.^22^, ^23^, ^24^, ^25^, ^26^ During the recovery period of COVID‐19, patients may experience long COVID, characterized by chest pain, fatigue, and even cognitive impairment, with risk factors considered to include old age, frailty, and female sex.^27^, ^28^ Although some therapies have been used for prevention and treatment, signs and symptoms may occur in patients with COVID‐19 and last for several months. A retrospective analysis suggested that neurological complications occur at a frequency of approximately 25%. Women infected with SARS‐CoV‐2 during pregnancy may have more severe long‐term COVID‐19 symptoms such as fatigue, myalgia, and olfactory disorders.^29^, ^30^ In addition, the discomfort, stress, and anxiety associated with COVID‐19 may induce mental health problems. Indeed, subjects with persistent COVID‐19 symptoms have been shown to have poorer mental health and health‐related quality of life, with longer recovery for women with persistent symptoms. People infected with SARS‐CoV‐2 are at an increased risk of anxiety and depression, and those who have been previously affected by these disorders may experience worsening illness. A national cohort survey revealed that adolescents present with multiple symptoms, with approximately 30% still experiencing fatigue, headache, and mental health problems 3 months after infection. Consequently, people diagnosed with anxiety or depression before infection with SARS‐CoV‐2 had higher rates of severe illness than those without mental disorders.^31^, ^32^, ^33^, ^34^

#### 2.2.2 COVID‐19 causes cognitive impairment

A recent systematic review of 13,232 patients with COVID‐19 revealed that approximately 22% developed cognitive impairment 12 weeks after COVID‐19 diagnosis. Cognitive impairment may be more serious when combined with impaired lung function and severe inflammation.^35^ Cognitive impairment is often accompanied by an abnormal psychological state, which affects the prognosis of patients and causes a chain reaction. A study involving 114 survivors of COVID‐19‐associated acute respiratory distress syndrome evaluated patients who were discharged from the intensive care unit and found that the rate of cognitive impairment at 3 months was 28%. These patients often have depression, anxiety, and stress disorders, all of which affect their quality of life.^36^ COVID‐19 may also induce brain structural changes. Indeed, Douaud et al.^37^ analyzed the differences in magnetic resonance imaging before and after SARS‐CoV‐2 infection and found evidence of brain structural alterations, including a greater reduction in global brain size and gray matter thickness. Similarly, sequencing data analysis of brain samples from patients with COVID‐19 and control individuals suggested inflammatory cell infiltration and broad cellular perturbations. The state of glial cells in COVID‐19 may be similar to that observed in neurodegenerative diseases, in which the synaptic signals of neurons are affected and may be involved in cognitive impairment.^38^ Elderly individuals are more prone to cognitive impairment due to aging and chronic comorbidities. Dementia and cognitive decline in the elderly may worsen mortality and convalescence complications after SARS‐CoV‐2 infection.^39^ Patients with Alzheimer's disease (AD) and advanced age are more likely to contract and die from COVID‐19.^40^ A survey of nursing home residents with a median age of 80 years found that SARS‐CoV‐2 infection caused cognitive impairment, malnutrition, and depression in the elderly. This highlights the importance of rehabilitation to improve the long‐term adverse effects of COVID‐19, such as cognitive impairment, thus reducing the burden of medical care.^41^

#### 2.2.3 Potential contribution mechanisms mediating COVID‐19‐related symptoms

The inflammatory response and immune dysfunction may play an important role in long COVID‐19. Inflammatory cells mediate chronic inflammation and tissue damage, leading to acute sequelae of COVID‐19 symptoms, which may have an underlying epigenetic mechanism. Targeting key inflammatory factors, such as IL‐6, may alleviate the persistent inflammatory burden caused by abnormal immune responses.^42^, ^43^ In addition, genome‐wide association studies have suggested that mutations in enzyme genes encoding related functions are involved in the mechanism of olfactory dysfunction induced by SARS‐CoV‐2 variants, further suggesting epigenetic changes.^44^ Moreover, metabolomics and proteomics studies of cerebrospinal fluid in neurological sequelae after acute SARS‐CoV‐2 infection have shown that sphingolipid metabolism disorder is associated with reduced inflammatory response, suggesting that metabolomic changes play a role in the repair process.^45^

**FIGURE 1:**
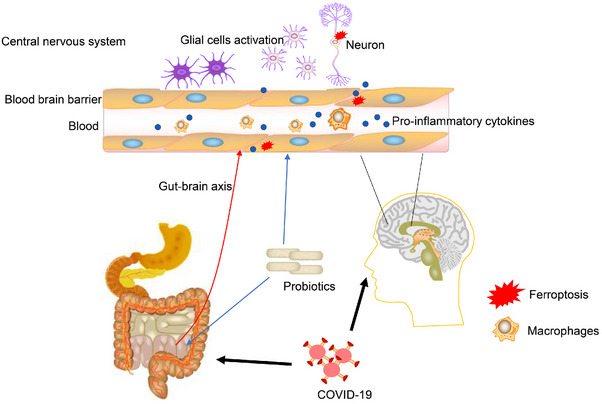
The process by which SARS‐CoV‐2 invades the brain, and the process by which probiotics regulate microbiota. The virus attacks the brain, causing intestinal dysfunction and neuroinflammation. Probiotic supplementation regulates the gut–brain axis and blood–brain barrier, thus alleviating symptoms by reducing glial cell activation and oxidative stress.

Elderly individuals show alterations in the intestinal barrier composition of the intestinal flora, which is accompanied by decreased immune function. These findings are crucial for understanding the effects of COVID‐19 on the brain in the acute phase and during long COVID. COVID‐19 causes changes in the blood–brain barrier (BBB) and the infiltration of inflammatory cells into the brain.^46^, ^47^ Hypoxia may also aggravate cognitive impairment in patients with COVID‐19. In a cross‐sectional investigation, Dondaine et al.^48^ divided 62 patients with COVID‐19 into two groups according to their hypoxia status. The researchers further explored cognitive function, and they found that hypoxia may be linked to cognitive impairment.^48^ Hypoxia may regulate cell metabolism and inflammation through HIF‐1α activation. Therefore, targeted improvement of hypoxia may regulate multiple organ functional status and relieve potential complications associated with hypoxia.^49^, ^50^ Neuroinflammation, BBB disruption, and neuronal cell changes have been observed in the hippocampus of humans and hamsters with COVID‐19. These findings may be related to neurogenesis, neuronal damage, and neurotransmitter abnormalities, which in turn contribute to cognitive impairment. Moreover, mice infected with SARS‐CoV‐2 showed persistent impairment of hippocampal neurogenesis, which is accompanied by loss of the myelin sheath.^51^, ^52^, ^53^ SARS‐CoV‐2 spike proteins or protein fragments are released and enter the brain during infection; this is accompanied by glial activation and synaptic reduction, which may be associated with memory loss and cognitive impairment.^54^ Overall, BBB damage, oxidative stress, neuroinflammation, hippocampal neuron dysfunction, hypoxia, and gut microbiota disorders may be involved in cognitive impairment in COVID‐19.^55^ A diagram of the virus attacking the brain, causing intestinal dysfunction and neuroinflammation is shown in Figure 1.

### 2.3 Vaccines against SARS‐CoV‐2 and variants

**FIGURE 2:**
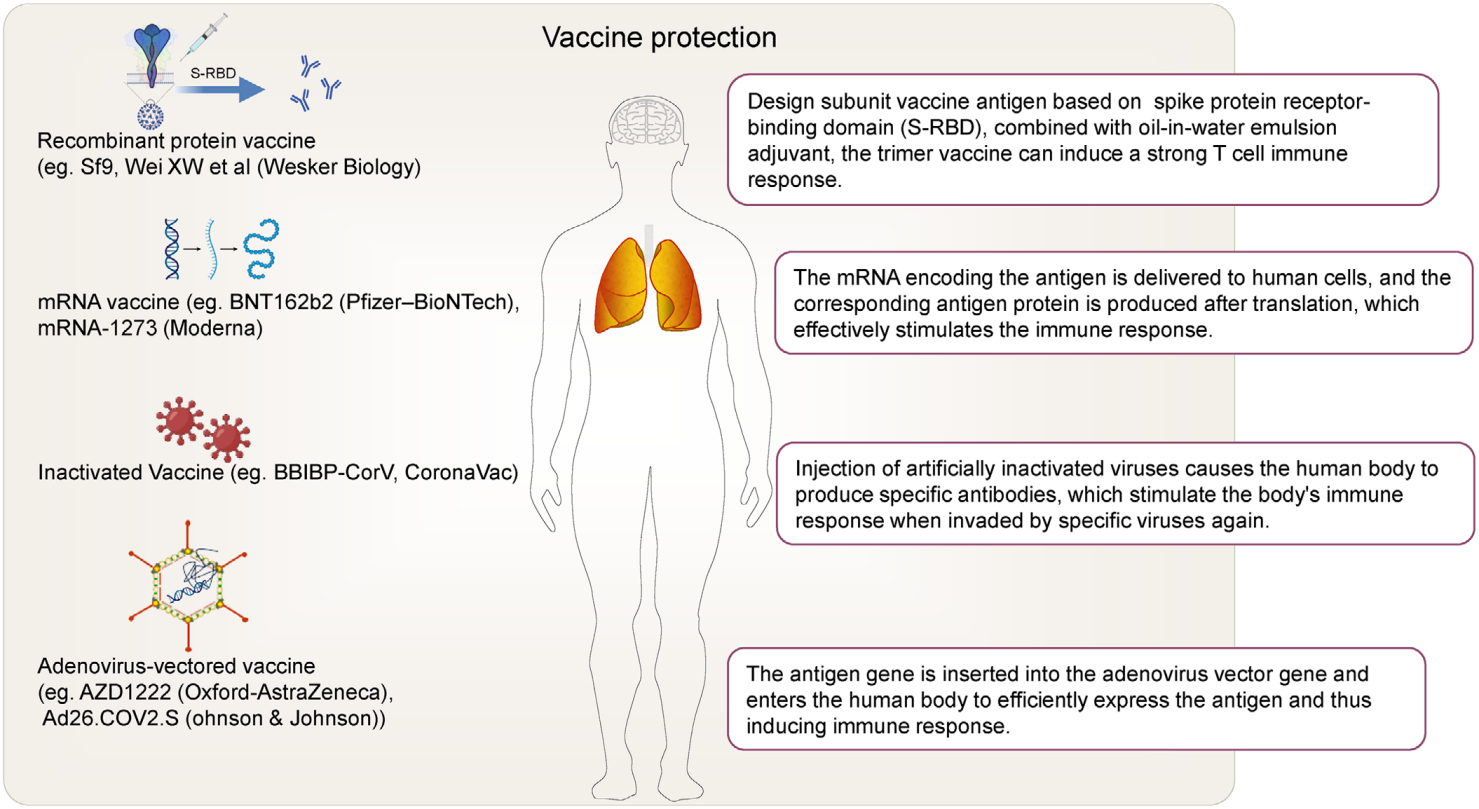
Different vaccines stimulate the body's immune response in different ways are shown. The four typical vaccines include recombinant protein vaccine, mRNA vaccine, inactivated vaccine, and adenovirus‐vectored vaccine.

Various vaccines have been used to prevent the COVID‐19. Different vaccines stimulate the body's immune response in different ways and have achieved significant preventive effects. Wei et al. found that serial vaccination with recombinant protein vaccines on the basis of existing vaccine immunity can induce a stronger immune response and increase the titer of neutralizing antibodies (Figure 2).^56^, ^57^, ^58^, ^59^, ^60^, ^61^, ^62^ However, the virus is constantly mutating, and there are still many COVID‐19 patients. SARS‐CoV‐2 invades cranial nerves and brain regions, affect immune function, and disrupt BBB. Elderly individuals are prone to cognitive impairment due to a weaker immune system and the breakdown of BBB.^63^ Vaccine significantly affected virus and pandemic trajectories. Previous infection with a SARS‐CoV‐2 variant provides some protection against symptomatic reinfection, and vaccines provide additional protection. The effectiveness of revaccination against confirmed SARS‐CoV‐2 infection decreases with time and increases with the third dose among adolescents, and strengthening vaccination could help reduce the burden on health care systems and adolescent morbidity. Access to an efficient health sector may influence vaccine decisions, and building a reasonably effective primary care system and ensuring a basic level of affordability for all should be at the head of pandemic preparedness strategies.^64^, ^65^, ^66^, ^67^ The vaccine may have additional protective effects in hippocampal neurogenesis. Indeed, it has been suggested that vaccine‐induced enhancement of hippocampal neurogenesis may offer protection against age‐related cognitive impairment and mental health conditions such as anxiety and depression in adults who have been vaccinated against SARS‐CoV‐2.^68^, ^69^ In addition, among adolescents, early vaccination in uninfected children who are exposed to the virus for the first time may enhance immunity against future variants, suggesting the long‐term effectiveness of vaccines.^70^

Different types of vaccines are associated with differences in mortality and adverse cardiovascular events. Adverse events following COVID‐19 mRNA vaccination include cardiovascular complications such as stroke, myocarditis, and thrombosis. A systematic review of confirmed cardiovascular complications after mRNA vaccines found that thrombosis, stroke, myocarditis, and pulmonary embolism were frequently reported with mRNA vaccines. The mean time from vaccination to first symptom onset was approximately 5 days.^71^ In general, the vaccine has greatly improved the symptoms of COVID‐19 patients, protected many susceptible people, and reduced the incidence of severe cases. Some potential vaccine‐related side effects have also caused anxiety in some people. Further research and evidence are required to improve the efficacy of the vaccine while reducing complications or side effects.^72^, ^73^

## 3 DYNAMIC CHANGES OF MICROBIOTA IN COVID‐19

Previous studies have shown that microbiomes play an important role in regulating host health and immunity. Microbiota alterations in COVID‐19 provide new insights into the potential pathophysiological process of COVID‐19‐related symptoms and complications. Here we summarize gut microbiota dysbiosis and the changes in vaccine‐associated microbiota and microbiota during acute infection and recovery.

### 3.1 Changes in microbiota during acute infection with SARS‐CoV‐2 and recovery

#### 3.1.1 Alterations in gut microbiota

As an important immunomodulatory body, the microbiota dynamically changes following SARS‐CoV‐2 infection, which is related to the symptoms. Compared with healthy controls, patients with COVID‐19 in the early stage of recovery exhibit decreased diversity and abundance of microbiota. Recovery does not immediately return the microbiota to normal levels, suggesting that patients with COVID‐19 have a significant gut microbiota imbalance that may persist for an extended period of time. A study conducted at multiple time points during hospitalization suggested significant dynamic changes in the fecal microbiome, characterized by the consumption of some beneficial commensal bacteria and the deficiency of beneficial microorganisms. This intestinal ecological disorder persisted after virus clearance, while some symptoms disappeared. Furthermore, correlation analysis suggested that the abundance of harmful bacteria, such as *Enterobacteriaceae*, was correlated with the severity of symptoms. COVID‐19 progression and severity are associated with changes in the gut microbiota following SARS‐CoV‐2 infection, which may be caused by changes in the abundance of key bacterial groups that are associated with host immune dysregulation.^10^, ^74^, ^75^, ^76^ SARS‐CoV‐2 recognizes and invades cells through ACE2 and may affect immune and metabolic functions. The ACE2 receptor is highly expressed on intestinal cells, which are an important site for virus entry into the intestine. Following invasion, SARS‐CoV‐2 replicates in intestinal epithelial cells, causing gastrointestinal symptoms. The entry of SARS‐CoV‐2 into the bloodstream can further cause excessive activation of platelets and inflammatory cytokines, resulting in disturbance of the intestinal microenvironment and loss of the gut–blood barrier, aggravating the changes in intestinal flora. Numerous symptoms may accompany the severity of the disease.^77^ The imbalance in gut microbiota may also be related to the imbalance in metabolite level, while the resulting inflammatory cytokine storm can cause overall immune disorders in the body. Viruses can easily invade the intestinal tract because of the high ACE2 expression in lung tissue and intestinal epithelial cells. The disruption of intestinal homeostasis during severe viral infection may be related to the progression and outcome of the disease, and the regulation of the gut–lung axis is critical to viral infection. The gut–lung axis involves ACE2, immune homeostasis, and dynamic regulation between the gut and lung. The human gut microbiota plays an important role in resisting viral infection. Changes in the abundance of gut microbiota or the concentration of its metabolites can cause immune disorders, and viruses can also regulate the homeostasis of the immune system.^78^, ^79^, ^80^

#### 3.1.2 Changes in the microbiota of the upper respiratory tract

According to previous studies, the gut microbiome interacts with the oropharynx. The microbial composition and function of the upper respiratory tract and gut are altered in patients with COVID‐19, and these changes may correlate with disease severity. The changes in upper respiratory tract flora show specificity compared with the intestinal flora imbalance, and the changes in microbial flora abundance in the upper respiratory tract may also mediate drug resistance by regulating metabolism and immunity.^81^ Gastrointestinal dysbiosis and related metabolic changes may also be associated with gastrointestinal symptoms. Indeed, patients with long COVID show differences in the abundance and function of oral and intestinal microbes and serum metabolites. Long‐term follow‐up and omics studies have found that patients with COVID‐19 and long‐term gastrointestinal symptoms have ectopic flora colonization from the gut to the mouth, which may be related to potentially harmful metabolites in the serum.^82^ In addition, lipidemic correlation analysis of nasopharyngeal and gut microbiota in hospitalized patients during the pandemic period found that the microbiome had metabolic effects on the host immune response, suggesting that related metabolites regulate the inflammatory state and internal environmental disorders in patients with COVID‐19. Nasopharyngeal microbiota may be associated with SARS‐CoV‐2 infection. Analysis of nasopharyngeal microbiota abundance in hospitalized patients with COVID‐19 and healthy controls found that some gut microbiota and nasopharyngeal microbiota participate in the host immune response.^83^, ^84^, ^85^

#### 3.1.3 Changes in microbiota and immune microenvironment

SARS‐CoV‐2 infection is associated with intestinal inflammation, intestinal barrier disruption, and changes in lipid metabolism. A decrease in short‐chain fatty acid (SCFA)‐producing bacteria is associated with infection. Further analysis suggested a strong correlation between some intestinal flora and the severity of SARS‐CoV‐2 infection and inflammatory indicators.^86^, ^87^, ^88^ Previous evidence has also shown that respiratory virus infection can cause an imbalance in gut microbiota, and diet, environmental factors, and genetic factors play an important role in the formation of the gut microbiota and immune response. In individuals with frailty and low immunity, such as the elderly, the diversity of intestinal flora is reduced, and the disorder of intestinal flora may be more significant.^89^ The intestinal flora involved in the synthesis and metabolism of SCFAs may change significantly during SARS‐CoV‐2 infection. An analysis of the intestinal microbiome in patients with COVID‐19 before and after the onset of symptoms revealed that compared with healthy controls, the function of intestinal flora in patients with severe COVID‐19 was significantly changed, and the abundance and synthesis ability of SCFA‐related bacteria were changed. A reduction in SCFA and l‐isoleucine metabolites was also observed in patients with COVID‐19 after remission. The concentrations of metabolites were significantly correlated with the blood inflammatory response, suggesting that related microorganisms mediate the host immune response, and related intestinal microbial pathways were identified.^90^ When the intestinal flora is dysregulated and the microenvironment is abnormal, immune‐related cytokines can further affect the immune and inflammatory responses in the lung through blood circulation. Recently, population‐based Mendelian randomization studies have provided new evidence for the causal relationship between COVID‐19 and changes in the gut microbiota. Multiple microbiotas were associated with SARS‐CoV‐2 infection and the disease severity, and hospitalization of patients with COVID‐19 led to an increase in the abundance of *Bacteroidetes* (*p* < 0.05).^91^ SARS‐CoV‐2 may also cause a high fever, although the physiological role of fever in the host's resistance to viral infection is unclear; however, the increase in body temperature may be accompanied by a change in the body's microbial flora, which may regulate the host's tolerance to the virus, thereby regulating the body's microenvironment in a form of feedback. High temperature exposure in mice can cause changes in the gut microbiota and microbiota‐related metabolites, such as the increased production of bile acids in an intestinal microbiota‐dependent manner. The amplification of signal cascades generated by deoxycholic acid produced by gut microbiota and its downstream receptor activation can inhibit viral replication and inflammatory cell infiltration and regulate human resistance to viral infection, suggesting that virus‐induced hyperthermia increases host viral tolerance in the form of changes in intestinal microbial abundance.^92^

The gut microbiome is closely related to disease progression. The probiotic *Bifidobacterium* is reduced in patients with severe COVID‐19. ACE2 may also play a complex regulatory role in SARS‐CoV‐2 infection and intestinal flora imbalance. Indeed, evidence suggests that intestinal flora can also regulate ACE2 expression and then regulate the gut–lung and gut–brain axis to mediate the dynamic interaction of the immune system. In elderly patients and those with severe COVID‐19 with intestinal flora imbalance, inflammation is significantly dysregulated and may be involved in intestinal flora imbalance and microenvironment disorder. Patients with significant intestinal flora imbalance have a poor prognosis, which is accompanied by a decrease in the abundance of butyrate‐producing flora, suggesting that changes in intestinal flora during hospitalization are related to the poor prognosis of patients with severe COVID‐19.^93^ Patients with COVID‐19 with immune and metabolic abnormalities, such as diabetes, also exhibit some specific common flora changes. The relative abundance of *Shigella* and *Bacteroides* is higher in patients with COVID‐19 with diabetes, suggesting that the imbalance in intestinal flora is increased and that dysregulated intestinal flora may also regulate metabolism and immunity.^94^ As discussed above, COVID‐19 changes the microbiota and the immune microenvironment in a closely related way.

### 3.2 Gut microbiota dysbiosis and symptoms

#### 3.2.1 Dysbiosis is involved in the symptoms of COVID‐19

Due to dysbiosis of the gut microbiota, metabolites have immunomodulatory effects on vaccine immunogenicity and also contribute to the occurrence and severity of COVID‐19 symptoms via modulating the entry of SARS‐CoV‐2 into the body and the resulting inflammatory response.^95^ The gut microbiota has also been linked to disease severity in COVID‐19. The analysis of intestinal flora showed that the abundance of antibiotic‐resistant *Enterobacteriaceae* in the gut of patients with COVID‐19 increased after virus infection. Animal experiments have also found that COVID‐19‐related symptoms and sequelae, such as pneumonia and cognitive impairment, occur in fecal transplantation mice 1−4 months after SARS‐CoV‐2 infection, indicating that the gut microbiota may directly contribute to COVID‐19‐related symptoms. Fecal transplantation from patients with COVID‐19 into germ‐free mice causes lung inflammation and cognitive impairment, indicating that the long‐term effects of the virus on intestinal flora directly mediate COVID‐19‐related symptoms and long COVID.^96^, ^97^ In addition, cohort studies on the association between gut microbiota and the severity of COVID‐19 symptoms have found that some gut microbiota are significantly associated with disease severity. Moreover, the abundance of actinobacteria can predict poor prognosis after infection, suggesting the feasibility of prediction models based on gut microbiota.^98^ Dysbiosis of gut microbiota is associated with increased susceptibility to the respiratory tract and immune microenvironment in the lung by regulating the gut–lung axis. Dysbiosis may also play an important role in central inflammation through the gut–brain axis to mediate central symptoms. Because the recovery of intestinal dysbiosis requires a certain period of time, the related symptoms mediated by intestinal dysbiosis may continue to affect patients with COVID‐19.^99^, ^100^ Most patients with COVID‐19 have mild symptoms and recover well, but some patients develop severe COVID‐19. A strong immune system balanced by anti‐inflammatory mechanisms is essential for recovery from SARS‐CoV‐2 infection. COVID‐19 is often accompanied by gastrointestinal symptoms and intestinal flora dysbiosis, mainly manifested by an increase in opportunistic pathogens and a decrease in beneficial symbiotic bacteria. A previous study has highlighted the correlation between dysbiosis and clinical symptoms of COVID‐19, and the gut microbiota is closely related to diarrhea and related gastrointestinal symptoms. Frail elderly people have higher disease severity and mortality, and the prognosis of COVID‐19 is worse in patients with gastrointestinal symptoms, which may be related to a more significant intestinal flora disorder.^101^, ^102^ The composition of the gut microbiota can also be altered by dietary and antibiotic environmental factors. Indeed, a prospective cohort study found that the composition of the gut microbiota changed with the course of COVID‐19 and was significantly correlated with cytokine levels, while the microbiota were significantly associated with poor prognosis. The gut microbiota may also affect the body's immune response by regulating the cytokine response and metabolic process, thereby affecting the prognosis of COVID‐19.^103^ Although the mechanism by which the gut microbiota mediates COVID‐19‐related symptoms remains controversial, abnormal immune activation mediated by dysbiosis, microbiota‐related specific metabolites, the gut–lung axis, and the gut–brain axis may be involved.

SARS‐CoV‐2 invades the respiratory tract and intestinal cells through the ACE2 receptor, leading to changes in intestinal flora and intestinal cell damage. Disorder of intestinal flora destroys the gut–blood barrier and triggers immune activation, which is associated with systemic symptoms.^77^ Multiomics association analysis revealed a potential regulatory relationship between intestinal flora and metabolites such as branched‐chain amino acids and the chemotactic response of inflammatory factors in patients with COVID‐19, and the intestinal flora may regulate lung and brain functions through metabolites. The severity of COVID‐19 is partly associated with host immune dysregulation, and Mendelian randomization studies have suggested that the gut microbiota is associated with susceptibility, hospitalization rate, and severity. Some patients with severe COVID‐19 have more significant dysbiosis of gut microbiota due to ICU admission, with decreased abundance of beneficial bacteria such as butyric acid microbes, and increased levels of C‐reactive protein. Therefore, changes in the gut microbiota during hospitalization may be associated with poor prognosis and increased 60‐day mortality in patients with severe COVID‐19.^104^, ^105^ The gastrointestinal tract may also be directly affected by the SARS‐CoV‐2 infection‐mediated immune response and the release of inflammatory mediators, which can lead to the chemotaxis of viruses from the respiratory epithelium into the gastrointestinal tract. The impairment of intestinal barrier function is the key factor leading to the imbalance in flora and the translocation of inflammatory substances, which in turn induces a stronger systemic immune response and leads to COVID‐19‐related sequelae. Multiple components of the intestinal immune system are affected by inflammatory mediators, chemotaxis of immune cells, and secretion of immunoglobulins in COVID‐19, leading to intestinal immune barrier dysfunction. The intestinal barrier can be maintained and self‐repaired to a certain extent by intestinal flora and metabolites. However, excessive activation of the immune response and immune microenvironment can cause further epithelial damage.^106^ A recent Mendelian randomization study on gut microbiota and COVID‐19 susceptibility and severity further suggested a correlation between gut microbiota dysbiosis and COVID‐19 symptoms, with some dysregulated flora, such as *Subdoligranulum* and *Lactobacillale* have been found to be associated with the severity of the disease (*p* < 0.05). These findings suggest that the gut microbiota may have a causal relationship with the severity of COVID‐19, thus providing new evidence and ideas for the study of the pathogenesis of SARS‐CoV‐2 mediated by gut microbiota.^107^, ^108^

#### 3.2.2 Potential causal relationship and pathophysiological processes between microbiota dysbiosis and COVID‐19‐related symptoms

**FIGURE 3:**
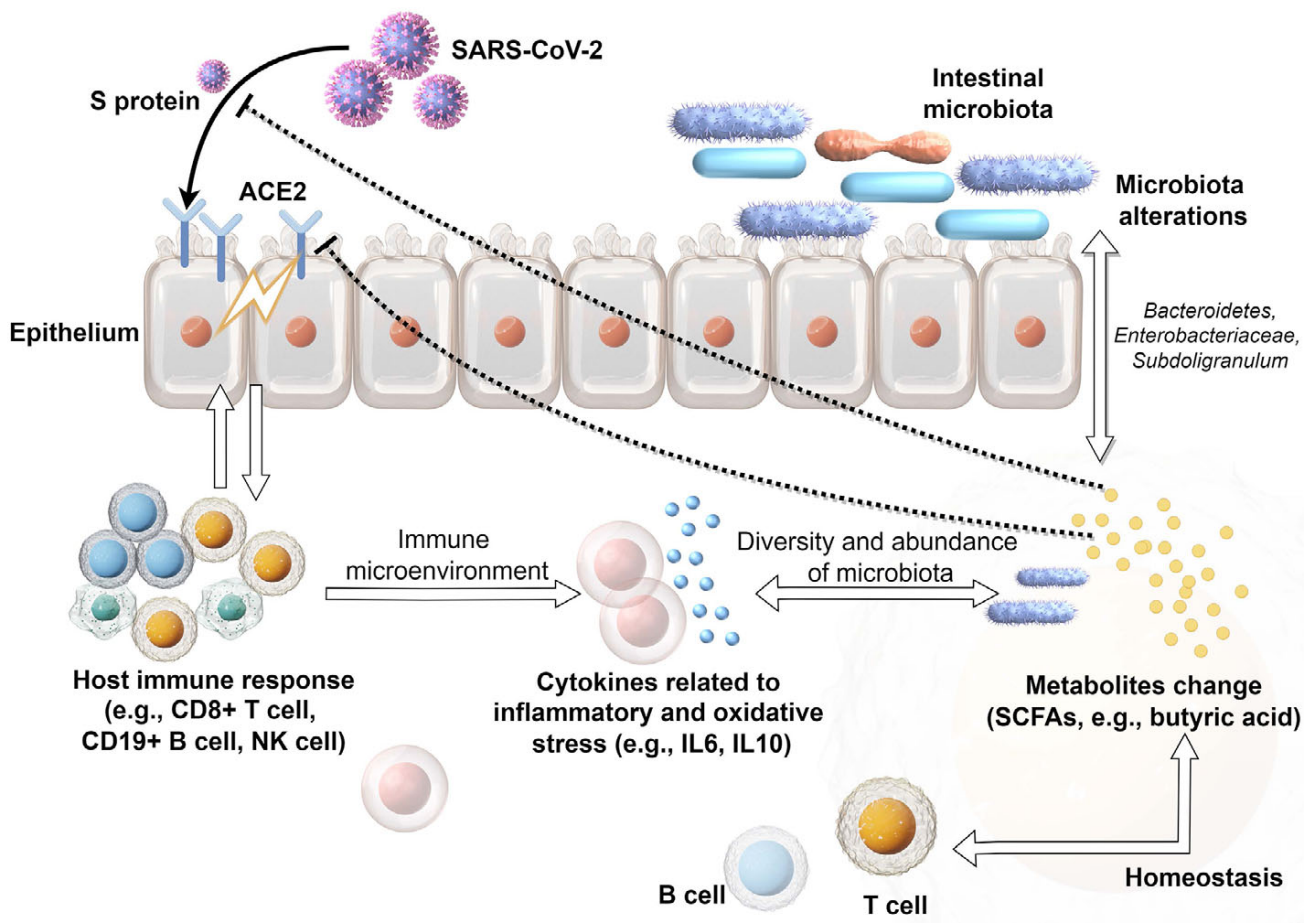
The potential causal relationship and processes between severe acute respiratory syndrome coronavirus 2 (SARS‐CoV‐2) and gut microbiota alterations. SARS‐CoV‐2 recognizes the angiotensin‐converting enzyme 2 (ACE2) receptor in the intestinal epithelium through S protein and triggers abnormal immune responses, such as activation of CD8+ T cells. The immune microenvironment interacts with microbiota alterations, which is accompanied by changes in short‐chain fatty acid (SCFA) metabolites. These metabolites may regulate the immune response, S protein activity, and ACE2 receptor expression.

Host immune disorders are closely related to intestinal microflora‐regulated immune homeostasis and intestinal microenvironment disorders. Subsequently, related bacteria produce bioactive metabolites, inflammatory factor responses, and changes in the immune microenvironment, which may promote the progression of COVID‐19. The bidirectional regulation between the gut microbiota and host immunity may play an important role in this process.^109^ In addition, SARS‐CoV‐2 may cause the disorder of gut microbiota, thereby increasing susceptibility to COVID‐19. The mechanisms by which the disturbance of gut microbiota increases susceptibility to COVID‐19 and associated symptoms are poorly understood. However, the imbalance in intestinal microbiota caused by COVID‐19 is known to affect the body's inflammatory and oxidative stress responses. Indeed, SCFAs are closely related to the inflammatory and oxidative state, which may be related to COVID‐19‐related complications. COVID‐19 alters the composition of gut microbiota and decreases the abundance of probiotic‐related bacteria. Metabolites produced by some bacteria can target the S protein of coronaviruses, thereby improving dysbiosis.^110^, ^111^ The reduced diversity of intestinal flora in patients with COVID can be accompanied by changes in circulating and intestinal SCFAs and an imbalance in circulating immune cell subsets. The proportions of CD19^+^ B, CD4^+^ T, CD8^+^ T, and NK cells of patients with COVID‐19 were significantly decreased, and the IL‐6 and IL‐10 levels were significantly increased, all of which were more prominent in critically ill patients. Further correlation analysis suggested that low SCFA levels in patients with COVID‐19 may be an important cause of lymphopenia.^112^ The influence of microbiota on the antiviral immune response includes the homing of dendritic cells and lymphocytes to the gut–lung axis. The composition of the gut microbiome influences ACE2 expression and inflammation.^113^ Working memory, attention, processing, and analysis abilities are significantly affected in some patients with COVID‐19 after infection, although the mechanisms underlying these chronic cognitive sequelae are currently unknown. SARS‐CoV‐2 can induce high levels of systemic cytokines, induce damage to the cerebral vessels and intestinal wall, and destroy the neurovascular unit of the brain and BBB. Pathogenic microbiota produce harmful substances and inflammatory molecules in the gut, which then infiltrate into the brain, eventually causing neuroinflammation and cognitive impairment.^114^ Gut–brain axis regulation is also reflected in some people with mental health disorders such as anxiety and depression, which are often accompanied by an abnormal abundance of gut microbiota, suggesting that intestinal flora disorder may also play a role in COVID‐19‐related anxiety and depression.^115^, ^116^ The potential causal relationship and processes between SARS‐CoV‐2 and gut microbiota alterations are shown in Figure 3.

**FIGURE 4:**
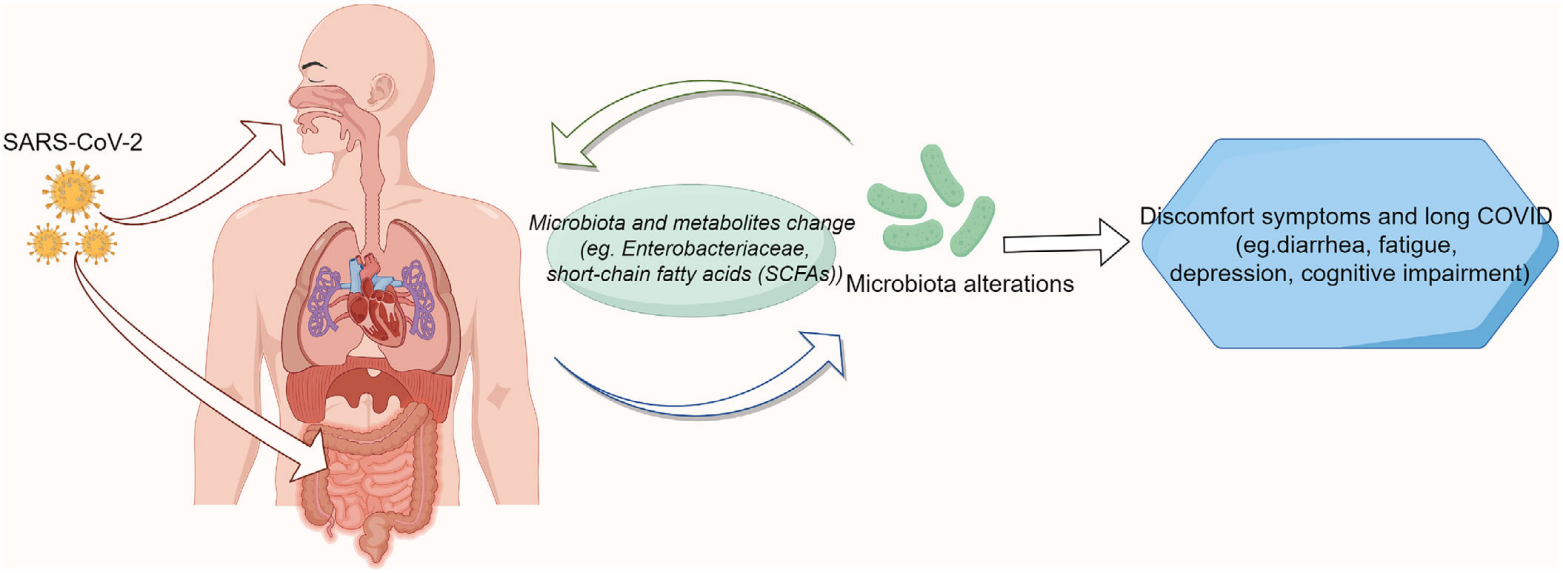
The changes in microbiota during SARS‐CoV‐2 acute infection and recovery. The abundance of gut microbiota (e.g. *Enterobacteriaceae*) and short‐chain fatty acids (SCFAs) changes after infection, which may be associated with symptoms such as diarrhea, fatigue, depression, and cognitive impairment.

A recent analysis using cortical sequencing data from patients with AD and COVID‐19 found significant changes in astrocyte and neuronal cell populations, suggesting that synaptic dysfunction, neuronal damage, and neuroinflammation jointly contribute to cognitive impairment in COVID‐19.^117^ Meanwhile, another study detected abnormal expression of AD biomarkers in the cerebrospinal fluid and blood of patients with COVID‐19, suggesting that brain microvascular damage plays a role in COVID‐19‐mediated cognitive impairment.^118^ Indeed, systemic infections that accompany severe cases of COVID‐19 trigger large increases in circulating chemokine and interleukin levels, which disrupt the BBB, leading to neuroinflammation and homeostasis imbalance.^119^ Glial activation and neuroinflammation may play an important role in cognitive impairment caused by COVID‐19. COVID‐19 leads to the upregulation of genes responsible for promoting abnormal synaptic function and migration and synaptic phagocytosis in microglia, which may result in cognitive impairment.^120^ Similarly, an analysis of brain pathology in elderly patients with cognitive impairment with COVID‐19 found a marked enhancement of microglia in the hippocampus.^121^ Another study involving brain tissue from 43 patients who died of COVID‐19 at a median age of 76 years detected the virus protein in 21 brains. However, the available evidence is insufficient to show that COVID‐19 directly causes central nervous system injury, indicating the complex mechanism of cognitive impairment caused by COVID‐19.^122^ Neuronal reactive autoantibodies are also known to be associated with COVID‐19‐related cognitive impairment. Alexopoulos et al.^123^ analyzed antibodies and albumin in the cerebrospinal fluid and serum of patients with COVID‐19 in a coma. The results suggested that cerebrospinal fluid SARS‐CoV‐2 antibodies may be related to BBB destruction, which in turn leads to inflammatory cell infiltration and neuroinflammation, the latter of which is closely related to cognitive deficits.^123^ Similarly, the study by Franke et al.^124^ included 11 patients with COVID‐19 with unexplained neurological symptoms, and analyzed blood and cerebrospinal fluid samples to detect antineuronal and antiglial autoantibodies. Notably, all participants showed antineuronal autoantibodies, which provided evidence for symptom interpretation and immunotherapy.^124^ In one study, antineuronal autoantibodies were detected in the blood and cerebrospinal fluid of more than half of the patients with COVID‐19 with cognitive impairment. However, further studies are needed to determine whether these antibodies play a direct role in cognitive impairment.^125^ The changes in microbiota during acute infection and recovery and the potential association between microbiota dysbiosis and symptoms are shown in Figure 4.

### 3.3 Vaccine‐associated microbiota changes

SARS‐CoV‐2 has a significant impact on the gastrointestinal tract by invading intestinal epithelial cells, which in turn causes intestinal flora imbalance and a series of cascade reactions. The inactivated SARS‐CoV‐2 vaccine has also been shown to induce changes in intestinal microbiota, with a previous study demonstrating that vaccinated versus unvaccinated individuals had significantly different abundance ratios and biological functions of intestinal flora, as determined by sequencing and abundance analysis.^126^, ^127^, ^128^ Vaccinated subjects showed a significant reduction in bacterial diversity, favoring an enterotype dominated by *Faecalibacterium* and gut microbiota enriched in *Faecalibacterium* and *Mollicutes*. Functional pathway enrichment analysis of microbiota abundance suggested that the transition of carbohydrate metabolism was positively correlated with vaccination, revealing that the abundance of bacteria related to neurodegenerative and cardiovascular diseases was significantly affected. These findings suggest that vaccination alters the composition of microbiota and its metabolites in the gut.^129^ The gut microbiota is associated with the vaccine response, which provides a basis for further exploring the regulation of the gut microbiota to improve vaccine efficacy. Newborns can obtain immune protection against SARS‐CoV‐2 through antibodies generated by the vaccine in breast milk, and vaccines can induce changes in the composition of the human breast milk microbiome, which may affect the antibody level. The richness and composition of the human breast milk microbiome change dynamically during the entire vaccination process, and microbiota are associated with high IgA levels.^130^ The gut microbiota may enhance or reduce the efficacy of vaccines through variations in metabolite composition. In addition to affecting gut microbiota abundance and composition, COVID‐19 vaccines may reduce the gut microbiota biodiversity.^131^ The efficacy of COVID‐19 vaccines also differs across individuals, and the gut microbiota may have an impact on its immunogenicity and thus its effectiveness. It appears that different microbiome components either enhance or reduce the efficacy of COVID‐19 vaccines, indicating a bidirectional relationship between the gut microbiome and the vaccine.^132^ The human gut microbiota and its metabolites may be involved in vaccine response. Vaccination is accompanied by changes in the composition and functional pathways of the gut microbiota, and the gut microbiota and its functional spectrum are related to the vaccine response. The serum SCFA levels have been shown to exhibit clear differences between high‐ and low‐response groups, while some SCFAs are positively correlated with antibody response. A dynamic change in the host immune system in conjunction with changes in the gut microbiota contributes to the production of antibodies against SARS‐CoV‐2.^133^, ^134^

**FIGURE 5:**
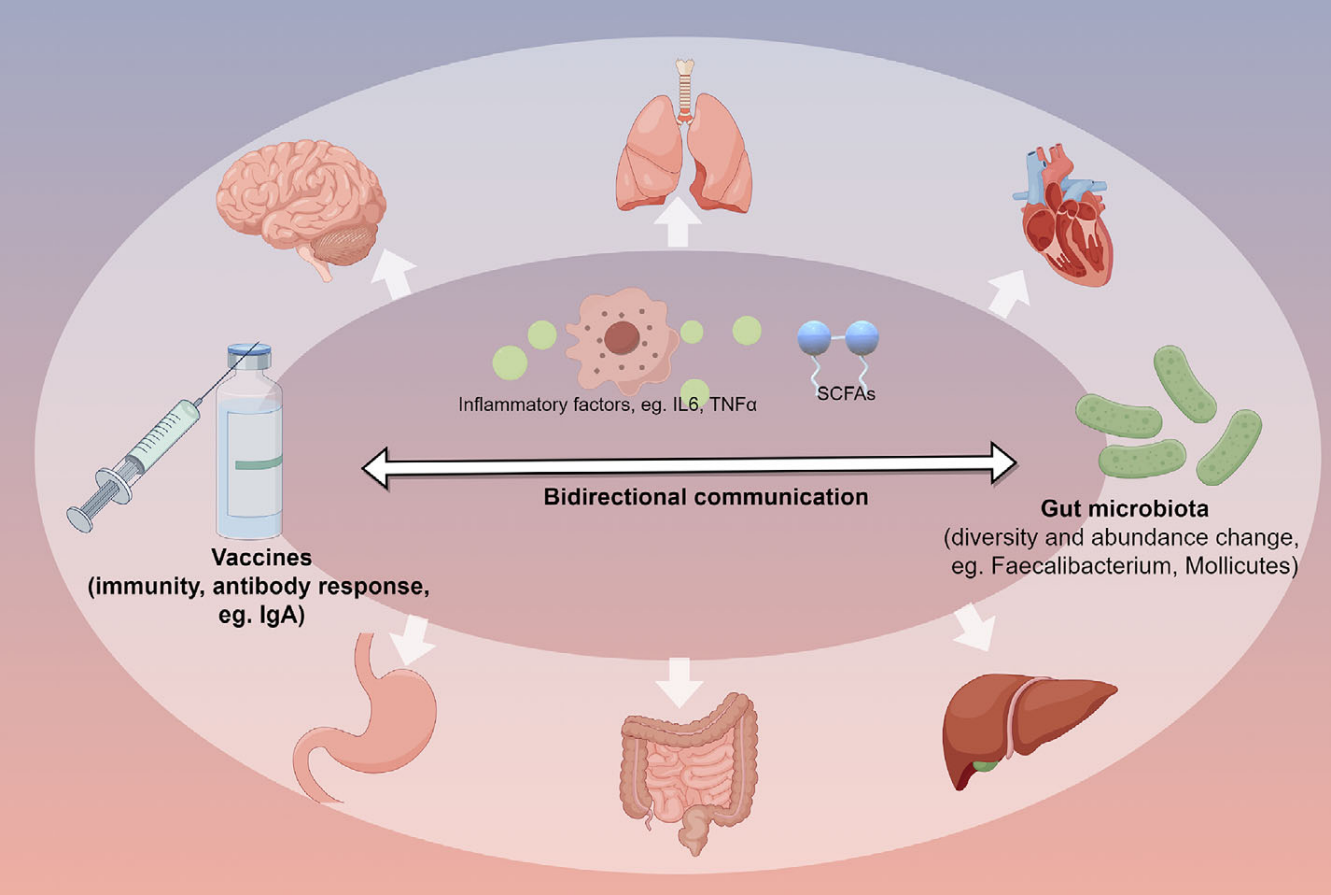
The changes in vaccine‐associated gut microbiota diversity. Vaccines may influence the diversity and abundance of the gut microbiota through modulation of the immune response. In turn, microbiota regulate the immune response to vaccines, inflammatory responses, and organ homeostasis. IL6, interleukin 6; TNFα, tumor necrosis factor‐α; SCFAs, short‐chain fatty acids.

Vaccines induce changes in the gut microbiota, the composition of which is associated with the immunogenicity and adverse events of SARS‐CoV‐2 vaccines, suggesting that the gut microbiota plays a key role in regulating the host immune response. *Prevotella copri* and *Megamonas* were found to be enriched in individuals with fewer adverse events following vaccination, suggesting that these bacterial groups play an anti‐inflammatory role.^135^ *Lactic acid bacteria* expressing foreign antigens have great potential as mucosal vaccines, while recombinant *Lactobacillus plantarum SK156* with the SARS‐CoV‐2 spike S1 epitope has been shown to induce humoral and cell‐mediated immune responses in mice. Mucosal vaccines can significantly alter the intestinal flora and derived metabolites of mice by regulating their composition and function, and increasing the abundance of beneficial intestinal bacteria, such as *Muribaculaceae, Mucispirillum*, and *Ruminococcaceae*. In addition, functional analysis of the gut microbiota revealed increased metabolic pathways for amino acids, carbohydrates, and vitamins. The concentrations of SCFAs, especially butyric acid, were also changed by mucosal immunity, further suggesting that vaccination affected the composition and metabolite levels of gut microbiota. In turn, the gut microbiome and its metabolites may influence the immunogenicity of SARS‐CoV‐2 vaccines.^111^, ^136^ Although the mechanism of this vaccine‐induced change in the microbiota and the bidirectional regulation of metabolites and inflammatory immune response caused by the changes in the microbiota has not been elucidated, exploring the relevant mechanisms provides new ideas for the optimization of vaccines and the selection of adjuvants. Vaccine‐associated microbiota changes and the potential bidirectional regulation are shown in Figure 5.

## 4 MICROBIOTA‐BASED IMPLICATIONS FOR THERAPEUTIC INTERVENTIONS

The microbiota alterations in COVID‐19 also have implications for probiotic‐based interventions. Probiotics are groups of organisms that are beneficial to the human body, such as *bifidobacteria* and *lactobacilli*. Probiotics may regulate the composition of the gut microbiota and affect immune function.^137^ We discuss the potential of probiotics to alleviate uncomfortable symptoms and cognitive impairment in COVID‐19 and improve vaccine potency.

### 4.1 Probiotic‐related treatment of COVID‐19

#### 4.1.1 Probiotics alleviate uncomfortable symptoms of COVID‐19

Recent bibliometric analysis has revealed the immunomodulatory properties of probiotics in patients with COVID‐19, highlighting their prospects for the adjuvant treatment of COVID‐19. We summarize the recently published clinical trials of probiotics for the treatment of COVID‐19 in Table 1.^138^, ^139^, ^140^, ^141^, ^142^, ^143^, ^144^ Supplementing probiotics may reduce inflammatory markers and regulate immune function. Approximately 26% of patients have cognitive impairment after infection, and this damage may persist for months. Moreover, elderly individuals are more likely to be affected by COVID‐19 because of frailty and comorbidities, and the impact is more severe.^145^, ^146^ A cross‐sectional study involving 1539 hospitalized patients with COVID‐19 and 466 healthy controls older than 60 years confirmed cognitive impairment 6 months after discharge, while the incidence of cognitive impairment was 12.45% at the 12‐month follow‐up.^147^ Comprehensive analysis suggested that severe COVID‐19 was associated with a higher risk of progressive cognitive decline (odds ratio: 19).^148^ However, there are few reports on the efficacy of probiotics in relieving cognitive impairment associated with COVID‐19.^149^, ^150^ Elderly patients with COVID‐19 are often accompanied by intestinal dysfunction and microbiota changes. Probiotics may regulate the gut–brain axis, which can be used to improve intestinal damage or drug‐related diarrhea caused by COVID‐19 treatment.^151^ Furthermore, it has been reported that probiotics promote mental flexibility, relieve stress, and significantly increase serum brain‐derived neurotrophic factor (BDNF) levels in healthy elderly patients, suggesting that they may improve cognitive function in elderly patients. Probiotics regulate the function of intestinal flora and inflammation in the elderly, which suggests a beneficial effect of probiotics on cognitive impairment associated with COVID‐19.^152^

**TABLE 1:** Clinical trials of probiotics in the treatment of COVID‐19.

Study/country	Participants (control/intervention)	Intervention	Follow‐up time	Side effect	Outcome	Conclusion
Vaezi et al. (2023)/Iran^138^	Adult COVID‐19 patients (38/38)	Multistrain probiotics (e.g., *Lactobacillus, Bifidobacterium, Streptococcus*), 10^18^ CFU per day, twice daily for 2 weeks	14 days	None	COVID‐19 clinical features, levels of proinflammatory IL‐6, CRP, ESR, and so on	Synbiotic adjuvant therapy can be effective to modulate inflammatory responses against COVID‐19.
Sandra et al. (2023)/Spain^139^	COVID‐19 subjects who required more than 48 h of hospital admission (99/101)	Multistrain probiotic (e.g., *Lacticaseibacillus, Bifdobacterium*), 10^9^ CFU, three times a day for no more than 14 days	During the hospital stay	NA	Mortality, digestive symptoms, hospital stays	It was effective in treating diarrhea associated with COVID‐19.
Richard et al. (2023)/Sweden and UK^140^	Healthy adults (78/81)	Probiotic product containing a minimum of 1 × 108 CFU of *Limosilactobacillus reuter*i DSM 17938 + 10 µg vitamin D3, twice daily for 6 months	6 months	Gastrointestinal complaints	Serum‐specific antibody titers, symptom duration and severity after infection and so on.	Supplementation with specific probiotics might improve the long‐term efficacy of mRNA‐based COVID‐19 vaccines via enhanced IgA response.
Pedro et al. (2022)/Mexico^141^	Adult symptomatic COVID‐19 outpatients (150/150)	*Lactiplantibacillus plantarum*, plus strain *Pediococcus acidilactici*, totaling 2 × 10^9^ CFU, for 30 days	30 days	None	Nasopharyngeal viral load, duration of both digestive and nondigestive symptoms, fecal microbiota	Probiotic supplementation significantly increased specific IgM and IgG. It is thus hypothesized this probiotic primarily acts by interacting with the host's immune system rather than changing colonic microbiota composition.
Saviano et al. (2022)/Italy^142^	Patients with COVID‐19 interstitial pneumonia (40/40)	*Bifidobacterium lactis LA 304*, *Lactobacillus salivarius LA 302*, and *Lactobacillus acidophilus LA 201*, probiotic mix twice a day for 10 days in addition to the standard COVID‐19 therapy	10 days	None	FECAL inflammatory markers (fecal calprotectin and CRP), any reduction in the need for nasal cannula or Ventimask oxygen support, the length of hospital stay	Supplementation with a mix of probiotics for 10 days in patients with COVID‐19 interstitial pneumonia significantly reduces inflammatory markers.
De boeck et al. (2022)/Belgium^143^	Unvaccinated COVID‐19 outpatients exhibiting mild‐to‐moderate symptoms (37/41)	Multispecies probiotic throat spray with *Lacticaseibacillus casei* AMBR2, *Lacticaseibacillus rhamnosus* GG, and *Lactiplantibacillus plantarum* WCFS1, for 14 days by spraying two puffs containing approximately 9.5 × 10^8^ CFU of lactobacilli multiple times a day	1 week	NA	Symptom severity, time to improvement, viral loads, antibodies, and the respiratory microbiome	It may reduce nasopharyngeal viral loads and acute symptoms.
Francesco et al. (2022)/Italy and UK^144^	COVID‐19 hospitalized patients (non‐ICU and not already receiving mechanical ventilatory support) (25/25)	Oral probiotic S. *salivarius* K12 plus standard of care, up to 14 days	14 days	NA	Biochemical parameters, fever, oxygen saturation level, need and length of oxygen therapy, the rate of progression to ICU and death	The adjuvant use of S. *salivarius* K12, an oral probiotic endowed with a well‐known capability to colonize the oral environment, improved the blood parameters and reduced the death rate.

COVID‐19, coronavirus disease 2019; CFU, colony forming units; IL‐6, interleukin‐6; CRP, C‐reactive protein; ESR, erythrocyte sedimentation rate; ICU, intensive care unit.

The potential benefits of probiotics for the prevention and treatment of COVID‐19 complications have attracted much attention. Elderly patients with COVID‐19 are prone to fatigue and cognitive impairment during recovery. Rathi et al.^153^ found that supplementation with probiotic complexes improves fatigue, attention, and memory. However, they did not focus on the improvement of cognition by probiotics. Mozota et al.^154^ found that probiotic dairy products significantly affected inflammatory factors such as IL8 and IL19 and increased the Barthel index and nutritional status. In further research, Mozota et al.^155^ evaluated the effect of *Ligilactobacillus salivarius* supplementation for 4 months on patients with COVID‐19 over the age of 75 years. The results showed that the inflammatory factor and cognitive status changed significantly, but the cognitive score did not, suggesting a potential benefit of probiotics in immunity regulation.^155^ Similarly, Vaezi et al.^138^ included 78 patients with COVID‐19 and randomly administered placebo or synbiotic capsules containing *Lactobacillus, Bifidobacterium*, and *Streptococcus* probiotics. After two daily interventions for 14 days, the level of interleukin 6 in the synbiotic supplementation group was significantly reduced.^138^ In addition, Catinean et al.^156^ suggested that patients receiving probiotic supplementation (five strains of bacillus; at least one month) may experience faster symptom resolution. Adjunctive therapy of *Limosilactobacillus reuteri* PBS072 and *Bifidobacterium breve* BB077 for 30 days improved the quality of sleep and mood, thus reliving stress.^157^ The above evidence supports that probiotic supplements reduce potential complications and disease burden.

Probiotics and their metabolites may restore the normal composition of intestinal flora by regulating the intestinal environment and intestinal barrier. Probiotic supplementation can reduce the symptoms of COVID‐19, such as diarrhea and dyspnea without obvious side effects.^158^ Commensal gut bacteria protect the gut environment and defend against viruses by promoting beneficial immune interactions. Regulation of gut microbiota may have a systemic antiviral effect in SARS‐CoV‐2 infection, which is related to the repair of intestinal barrier and anti‐inflammation. In addition, the use of probiotics can reduce the susceptibility of the population to respiratory virus infections such as COVID‐19, while regulating the immune response to improve the potency of vaccines. Probiotics mainly regulate the microbiota and immune system by regulating the innate system response and the production of anti‐inflammatory cytokines, thereby regulating the inflammatory and oxidative stress state of the body, and may regulate pneumonia and related symptoms.^159^, ^160^ A meta‐analysis found that probiotics shortened the length of hospital stay, recovery time, and reduced the risk of death, suggesting the potential of probiotics as an adjuvant therapy to reduce the risk of death and symptoms in COVID‐19.^161^ Based on the gut–lung axis theory, there is a bidirectional interaction between gut microbiota and lung. Probiotics reduce serum C‐reactive protein levels, shorten the length of hospital stay, and further improve respiratory symptoms through the gut–lung axis. In addition, probiotics can not only regulate the composition of intestinal flora and the concentration of related metabolites, but also affect the generation and function of Treg cells to further regulate immune function. Probiotics deserve more attention in regulating gut microbiota, maintaining intestinal homeostasis and serving as an antiviral mechanism.^162^, ^163^, ^164^

Studies of probiotic therapy as postexposure prophylaxis for COVID‐19 also suggest a potential protective effect. One study included 182 participants with close exposure to COVID‐19; it included a follow‐up for analysis of symptoms and the fecal microbiome after 28 days of continuous probiotic supplementation. *Lacticaseibacillus rhamnosus GG* group had fewer disease symptoms than placebo (26.4 vs. 42.9%), suggesting that probiotic prophylaxis is associated with a lower incidence of COVID‐19 symptoms and changes in the gut microbiome.^165^ Probiotics enhance the immunity of patients with SARS‐COV‐2, which in turn causes inflammation and increased cytokine secretion. Probiotics can also improve lung function in patients with SARS‐COV‐2 by regulating ACE2. Probiotic intake is associated with reduced levels of inflammatory markers, which can modulate inflammasomes to stimulate immune responses to block viral invasion and replication. Probiotics may also target SARS‐CoV‐2 by blocking viral invasion and replication and stimulating immune responses by modulating inflammasomes. In addition, some bacteria have relatively direct antiviral properties. For example, *lactic acid bacteria* can stimulate the host's innate antiviral immune defense system to produce antiviral peptides to prevent viral replication or invasion. Adherence of the SARS‐CoV spike protein to the surface of *B. breve* may trigger the host immune response and trigger antibody production.^166^, ^167^, ^168^ Probiotics also improved symptoms and viral clearance in outpatients with COVID‐19, with supplementation reducing nasopharyngeal viral load, pulmonary infiltrates, and duration of unwell symptoms, as compared with placebo. Probiotic supplementation significantly increased COVID‐19‐specific IgM and IgG. However, probiotics did not significantly affect the composition and abundance of gut microbiota in some individuals, suggesting that probiotics can also directly communicate with the body's immune system to play an immunomodulatory role.^141^ Given the ongoing effects of neuroinflammation caused by COVID‐19 and its related anxiety, depression, and cognitive impairment, probiotic‐mediated regulation of the gut–brain axis represents a novel idea for targeted intervention. Indeed, supplementation with probiotics *Lactiplantibacillus* can improve mood and sleep quality and protect cognitive function.^169^

#### 4.1.2 Probiotics may improve cognitive impairment in patients with COVID‐19

**FIGURE 6:**
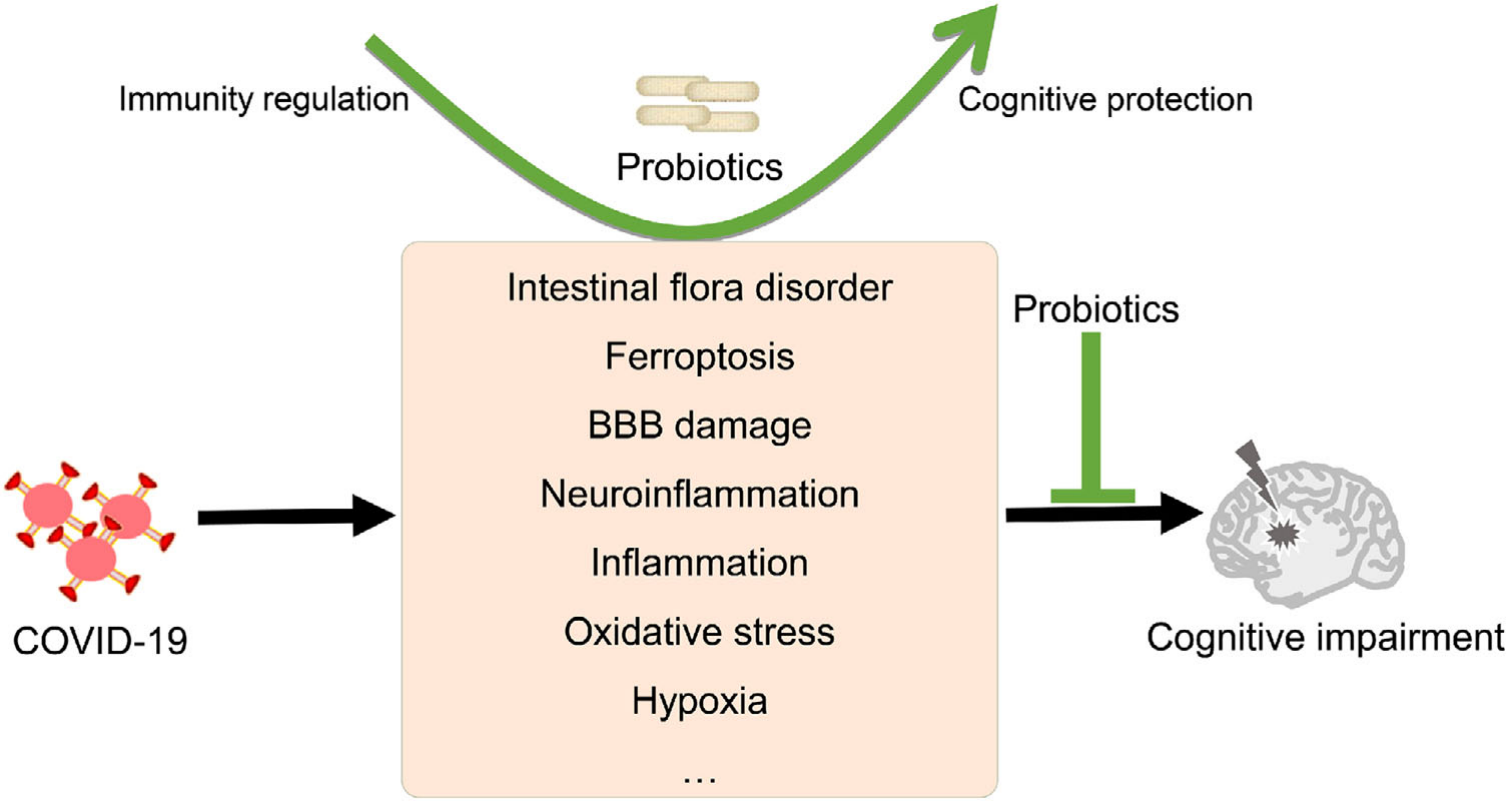
Potential mechanism of probiotics in the treatment of cognitive impairment caused by coronavirus disease 2019 (COVID‐19). Cognitive impairment may be alleviated by probiotics through regulating microbiota imbalance, ferroptosis, the blood–brain barrier (BBB), neuroinflammation, oxidative stress, and hypoxia.

The gut and brain are bidirectionally connected and regulate each other via the gut–brain axis. Using probiotics can regulate the immune response and inflammation to ameliorate BBB destruction, which plays a role in AD.^170^ The frail elderly may have more severe symptoms after SARS‐CoV‐2 infection, which will aggravate any existing cognitive impairment. Probiotic supplementation has been shown to significantly change the levels of tryptophan metabolism‐related metabolites in the elderly, among which indole‐3‐propionic acid (IPA) increased by 1.91 times and was positively correlated with serum BDNF levels. In vitro, IPA was found to increase BDNF levels in neurons and decrease TNF‐α levels in microglia, which further suggests the role of microbial metabolites in regulating neuroinflammation and cognition.^171^ Combined with machine learning algorithms and data model analysis, it was found that probiotic supplementation could regulate the anti‐inflammatory effect through the enhancement of bifidobacterium and related metabolism, which promoted the recovery of patients and the improvement of discomfort symptoms, suggesting its potential for treating long COVID.^172^ COVID‐19 can invade the brain and cause damage to the BBB, activating microglia in the hippocampus and ultimately contributing to cognitive decline. However, a prospective observational cohort study focused on the correlation between long‐term cognitive function in patients with severe COVID‐19 and anti‐inflammatory treatment found that the treatment did not significantly affect long‐term cognitive function. The study included 96 patients who were followed up for 6 months after hospital discharge.^173^ These findings may be due to the small sample size or short follow‐up time, but may also be due to its complex pathophysiology. In this situation, whether probiotic adjuvant therapy may improve the regulation of neuroinflammation is a crucial consideration for future investigations. The potential cognitive benefits of probiotic adjuvant therapy are worth looking forward to.^174^ A recent clinical cross‐sectional study demonstrated a correlation between alterations in the gut microbiome composition of patients with AD and β‐amyloid and tau pathological biomarkers, suggesting that changes in the gut microbiome may occur early in the disease process and have an association with cognitive impairment. Probiotics may mitigate COVID‐19‐induced cognitive impairments through the regulation of neuroinflammation, modulation of the gut microbiota, and gut homeostasis.^175^, ^176^ Overall, probiotics have important research potential and value in improving COVID‐19‐related cognitive impairment in the elderly, potentially via their complex regulation of various physiological processes (Figures 1 and 6).

### 4.2 Adjuvant improve vaccines potency

Gut microbiota metabolites can enter the circulation and play an immunomodulatory role throughout the whole body. Moreover, these molecules are recognized by immune cells from the host, which can trigger or regulate different responses and affect the efficacy of vaccines. Recent studies have suggested that modulating the composition or abundance of gut microbiota may be a means to achieve a more effective protective immune response. Notably, there may be an association between better vaccine response and specific microbiota. The use of probiotics in combination with vaccines indicates that bacterial components can also be used as adjuvants to improve and optimize the response to respiratory virus vaccines.^177^, ^178^ Interindividual differences in efficacy may be partly due to robust immune responses induced by mRNA vaccines targeting the SARS‐CoV‐2 spike protein and changes in the composition and abundance of gut microbiota that affect vaccine immunogenicity. Indeed, the levels of antispike IgG measured before and 1 week after vaccination correlated with the diversity of gut microbiota, which normally correlates with vaccine immunogenicity. Functional pathway enrichment analysis suggested that SCFA metabolism and sulfur metabolism‐related pathways may be involved, as well as being positively correlated with IgG levels. These results confirm that the gut microbiota composition influences the immunogenicity of SARS‐CoV‐2 mRNA vaccines.^179^ The role of the gut microbiome in modulating the durability of immunity against COVID‐19 vaccines remains unclear. Gut microbiota sequencing and blood analysis at baseline, 1 month, and 6 months after vaccination revealed that multiple inflammatory factors, chemokines, and antibodies were associated with gut microbiota and metabolites, and that the abundance of some microbiota was associated with higher antibody levels at 6 months, suggesting that microbiota adjuvants may prolong the durability of the immune response to the SARS‐CoV‐2 vaccine.^180^

The course of the immune response after vaccination in partially immunocompromised patients is helpful for studying vaccine response. In immunosuppressed patients with IBD, gut microbiota and metabolomics analysis demonstrated that the diversity of gut microbiota in sublevel responders was significantly lower, and that the abundance of some microbiota such as *Cholangiella* was associated with better serological response, suggesting that gut microbiota is involved in different serological responses after SARS‐CoV‐2 vaccination. Regulating the composition and abundance of the microbiota may affect the efficacy of the vaccine.^181^ Aging leads to major changes in the composition and function of the gut microbiome, including decreased diversity. The immunogenicity of COVID‐19 vaccines is affected by the composition of the gut microbiota, and the immune response to COVID‐19 vaccines decreases with age. The microbiome may be an important determinant of vaccine immunity, but the mechanisms of abnormal cellular function that influence the aging process and vaccine response are incompletely understood. The imbalance in gut microbiota in the elderly is closely related to immune senescence and gut microbiota. The intervention of probiotics targeting gut microbiota may improve the problem of reduced immune response in frail individuals.^182^, ^183^ Furthermore, vaccine‐induced immune responses may vary greatly among individuals, and vaccines are less immunogenic in populations at the highest risk of infectious diseases. The composition and function of the gut microbiota may serve as key factors regulating the immune response to vaccines. Gut microbiota disorder determines postvaccination immune efficacy, particularly susceptibility to SARS‐CoV‐2, and the severity of infection. Microbiota can comprehensively regulate the immune status of the body by regulating the levels of metabolites and inflammation, and can affect the functional activities of a variety of enzymes. Targeting the gut microbiota can affect the acquired immunity of secreted immunoglobulins and the microbiome to stimulate local gut immune responses. In addition, SARS‐CoV‐2 replication requires certain key enzymes, and regulation of the abundance of bacterial flora and its metabolite concentrations by probiotics may affect related processes and improve vaccine efficacy.^184^, ^185^, ^186^ The research and use of SARS‐CoV‐2 intranasal spray vaccine have attracted increasing attention. Considering the characteristics of upper respiratory tract flora changes after virus infection and its potential function in regulating the virus and immune microenvironment, further exploration of the role of flora and the mechanisms of immune regulation is imperative for developing and using related vaccines.^187^

### 4.3 Diagnosis and others

Based on the correlation between SARS‐CoV‐2 infection and changes in the abundance of intestinal microbiota in patients at various stages of infection, COVID‐19 has been found to cause a decrease in microbiota diversity, resulting in a reduction in butyrate‐producing bacteria. These changes also indicate immune dysregulation and are associated with some discomfort symptoms and poor prognosis. These findings suggest that the detection and biomarker analysis of intestinal flora after SARS‐CoV‐2 infection can be used as a therapeutic or intervention target to promote the rapid recovery of patients.^188^, ^189^ In addition, the intestinal barrier composition of the intestinal flora of the elderly changes, which is accompanied by decreased immune function. Probiotics regulate the composition of intestinal flora, affect intestinal sugar metabolism and vitamin synthesis, and in turn improve the overall inflammatory state.^190^ A retrospective analysis found that supplementation with probiotics for elderly individuals over the age of 65 years can regulate the levels of inflammatory factors such as IL‐8 and Il‐10, suggesting that probiotics may regulate inflammation and oxidative stress. Fermented lactic acid bacteria containing the glutamate decarboxylase gene may mediate the intestinal–brain axis through the neurotransmitter γ‐aminobutyric acid to improve cognitive impairment in elderly patients.^191^ In addition, surgery may cause intestinal flora disorders and BBB destruction, which are obviously related to postoperative delirium and postoperative cognitive impairment. Furthermore, supplementation with probiotics improved neuroinflammation and cognitive impairment in surgery.^192^, ^193^ Yang et al.^194^ found that probiotic preparations improved memory impairment in aging mice. The mechanism may be related to the repair of intestinal barrier and BBB functions, as well as the regulation of interleukin and tumor necrosis factor‐α, suggesting that probiotic therapy can target the intestinal–brain axis to improve cognition.^194^ Furthermore, there is evidence that probiotics improve inflammation, oxidative stress, and flora polymorphism in patients with AD and repair the BBB to a certain extent, thus improving cognitive decline. Moreover, no significant side effects were observed in the treatment, suggesting that probiotics have wide application prospects in AD treatment.^195^ A randomized controlled pilot trial comparing the effects of probiotics on cognitive function in patients with fibromyalgia also found that probiotics improve patients’ decision‐making ability to a certain extent, providing further evidence for probiotic supplementation in treating cognitive impairment.^196^ The potential pathways of cognitive impairment include inflammation, oxidative stress, BBB destruction, and the intestinal–brain axis. Probiotics may regulate these processes to improve cognitive impairment in AD. It has been reported that anti‐inflammatory bacteria (*Bifidobacterium, Faecalibacterium*) and inflammation‐associated microbiota (*Streptococcus, Actinomyces*) change significantly during COVID‐19. Probiotics such as *Limosilactobacillus fermentum* and *L. rhamnosus* may modulate intestinal dysbiosis and consequently relieve proinflammatory response.^75^, ^197^ Gut microbiota metabolites are closely related to host immune function. Therefore, probiotics may regulate the body's immune response by regulating flora balance and metabolism. Improvement of the inflammatory response caused by COVID‐19 is conducive to recovery. Ferroptosis, a type of cell death caused by iron‐dependent lipid peroxidation, has gradually attracted attention in the complications of COVID‐19.^198^ Supplementation with probiotics can also regulate the iron death process. It has been reported that glutathione peroxidase 4 (GPX4) inactivation, iron metabolism changes, and reactive oxygen species peroxidation upregulation are unique signs of COVID‐19. COVID‐19 may cause the iron‐mediated death of multiorgan cells, leading to multiorgan damage. Ferroptosis plays an important role in COVID‐19‐related myocarditis and lung injury.^199^, ^200^ Ferroptosis characterized by lipid peroxidation and high glutathione consumption is likely to mediate COVID‐19‐related brain injury, suggesting that iron death intervention may be used as a novel treatment for brain injury. Targeted therapy directed by probiotics and their metabolites provide novel ideas for the treatment of these diseases with comorbid mechanisms, which is worthy of further exploration.^201^, ^202^, ^203^

## 5 CONCLUSION AND PROSPECTS

Evidence suggests that COVID‐19 may be accompanied by microbiota alterations, and dysbiosis of the gut microbiota contributes to COVID‐19 symptoms and COVID‐19 development through metabolites and immune responses. A probiotic‐based intervention may improve COVID‐19 symptoms by regulating the gut microbiota and systemic immunity. However, further evidence is required to establish a causal relationship between changes in microbiota (e.g., *Bacteroidetes, Enterobacteriaceae*, and *Subdoligranulum*) and symptoms associated with COVID‐19. In addition, probiotic supplements such as *Lactobacillus, Bifidobacterium*, and *Streptococcus* may regulate intestinal immunity and intestinal barrier function through specific molecular mechanisms.

Probiotics may also be used as adjuvants to improve vaccine efficacy and prepare for rapidly changing viruses and the next pandemic. Indeed, a significant reduction in bacterial diversity has been reported among vaccinated subjects. Microbiome components appear to either enhance or reduce the efficacy of COVID‐19 vaccines, indicating a bidirectional relationship. The mechanism by which probiotics and SCFA metabolism affect IgG levels, and the specific dose and effect, are worthy of further investigation.

The role of probiotics in cognitive protection and immune regulation also offers insights into the treatment of aging, AD, and other diseases. Probiotics and their metabolites may be used to target microbiota and regulate ferroptosis and GPX4 to provide solutions to treat diseases with comorbid mechanisms. In addition, the pathophysiological processes of neuroinflammation and the gut–brain axis regulated by different probiotics and SCFAs require further study. Well‐designed clinical trials are urgently needed to enhance content credibility.

## AUTHOR CONTRIBUTIONS

Y. Q., G. C., and T. Z. conceived and designed this project. Y. Q., C. M., and G. C. wrote the manuscript. L. C., W. Y., G. C., and T. Z. checked and amended the manuscript. All authors contributed to the article, read, and approved the final manuscript.

## CONFLICT OF INTEREST STATEMENT

All authors declare no conflict of interest.

## ETHICS STATEMENT

Not applicable.

## Data Availability

Not applicable.

## References

[1] Worldometers.info . COVID‐19 Coronavirus Pandemic. Published February 6, 2024. Accessed February 6, 2024. https://www.worldometers.info/coronavirus/

[2] Bowe B , Xie Y , Al‐Aly Z . Postacute sequelae of COVID‐19 at 2 years. Nat Med. 2023;29(9):2347‐2357. 37605079 10.1038/s41591-023-02521-2 PMC10504070

[3] Thaweethai T , Jolley SE , Karlson EW , et al. Development of a definition of postacute sequelae of SARS‐CoV‐2 infection. JAMA. 2023;329(22):1934‐1946. 37278994 10.1001/jama.2023.8823 PMC10214179

[4] Wesselingh R . Prevalence, pathogenesis and spectrum of neurological symptoms in COVID‐19 and post‐COVID‐19 syndrome: a narrative review. Med J Aust. 2023;219(5):230‐236. 37660309 10.5694/mja2.52063

[5] Chee YJ , Fan BE , Young BE , Dalan R , Lye DC . Clinical trials on the pharmacological treatment of long COVID: a systematic review. J Med Virol. 2023;95(1):e28289. 36349400 10.1002/jmv.28289 PMC9878018

[6] Lui G , Guaraldi G . Drug treatment of COVID‐19 infection. Curr Opin Pulm Med. 2023;29(3):174‐183. 36917228 10.1097/MCP.0000000000000953 PMC10090306

[7] Ammirati E , Moslehi JJ . Diagnosis and treatment of acute myocarditis: a review. JAMA. 2023;329(13):1098‐1113. 37014337 10.1001/jama.2023.3371

[8] Malik P , Patel K , Pinto C , et al. Post‐acute COVID‐19 syndrome (PCS) and health‐related quality of life (HRQoL)‐A systematic review and meta‐analysis. J Med Virol. 2022;94(1):253‐262. 34463956 10.1002/jmv.27309 PMC8662132

[9] Liu Q , Mak J , Su Q , et al. Gut microbiota dynamics in a prospective cohort of patients with post‐acute COVID‐19 syndrome. Gut. 2022;71(3):544‐552. 35082169 10.1136/gutjnl-2021-325989

[10] Zuo T , Zhang F , Lui G , et al. Alterations in gut microbiota of patients with COVID‐19 during time of hospitalization. Gastroenterology. 2020;159(3):944‐955. e8. 32442562 10.1053/j.gastro.2020.05.048 PMC7237927

[11] Yeoh YK , Zuo T , Lui GC , et al. Gut microbiota composition reflects disease severity and dysfunctional immune responses in patients with COVID‐19. Gut. 2021;70(4):698‐706. 33431578 10.1136/gutjnl-2020-323020 PMC7804842

[12] Cheong KL , Chen S , Teng B , Veeraperumal S , Zhong S , Tan K . Oligosaccharides as potential regulators of gut microbiota and intestinal health in post‐COVID‐19 management. Pharmaceuticals (Basel). 2023;16(6):860. 37375807 10.3390/ph16060860 PMC10301634

[13] Markov PV , Ghafari M , Beer M , et al. The evolution of SARS‐CoV‐2. Nat Rev Microbiol. 2023;21(6):361‐379. 37020110 10.1038/s41579-023-00878-2

[14] Kodsi IA , Rayes DE , Koweyes J , et al. Tracking SARS‐CoV‐2 variants during the 2023 flu season and beyond in Lebanon. Virus Res. 2024;339:199289. 38036064 10.1016/j.virusres.2023.199289 PMC10704499

[15] Zhang X , Deng X , Zhang L , et al. Single‐cell RNA sequencing analysis of lung cells in COVID‐19 patients with diabetes, hypertension, and comorbid diabetes‐hypertension. Front Endocrinol (Lausanne). 2023;14:1258646. 38144556 10.3389/fendo.2023.1258646 PMC10748394

[16] Mei S , Zou Y , Jiang S , et al. Highly potent dual‐targeting angiotensin‐converting enzyme 2 (ACE2) and Neuropilin‐1 (NRP1) peptides: a promising broad‐spectrum therapeutic strategy against SARS‐CoV‐2 infection. Eur J Med Chem. 2024;263:115908. 37981444 10.1016/j.ejmech.2023.115908

[17] Patton MJ , Gaggar A , Might M , Erdmann N , Orihuela CJ , Harrod KS . Community‐acquired bacterial coinfections and COVID‐19. Physiol Rev. 2024;104(1):1‐21. 37589392 10.1152/physrev.00010.2023

[18] Dai S , Cao T , Shen H , et al. Landscape of molecular crosstalk between SARS‐CoV‐2 infection and cardiovascular diseases: emphasis on mitochondrial dysfunction and immune‐inflammation. J Transl Med. 2023;21(1):915. 38104081 10.1186/s12967-023-04787-z PMC10725609

[19] Hon K , Leung A , Leung K , Wong A . Impact of “long covid” on children: global and Hong Kong perspectives. Curr Pediatr Rev. 2024;20(1):59‐65. 36281870 10.2174/1573396319666221021154949

[20] Yisimayi A , Song W , Wang J , et al. Repeated Omicron exposures override ancestral SARS‐CoV‐2 immune imprinting. Nature. 2024;625(7993):148‐156. 37993710 10.1038/s41586-023-06753-7 PMC10764275

[21] Gulliford MC , Steves CJ . Access to COVID‐19 vaccination and COVID‐19‐related hospital admissions and mortality. Lancet. 2024. S0140‐6736(23)02622‐02623. 10.1016/S0140-6736(23)02622-3 38237626

[22] Alkodaymi MS , Omrani OA , Fawzy NA , et al. Prevalence of post‐acute COVID‐19 syndrome symptoms at different follow‐up periods: a systematic review and meta‐analysis. Clin Microbiol Infect. 2022;28(5):657‐666. 35124265 10.1016/j.cmi.2022.01.014 PMC8812092

[23] Choi BY , Grace AR , Tsai J . Heterogeneity of COVID‐19 symptoms and associated factors: longitudinal analysis of laboratory‐confirmed COVID‐19 cases in San Antonio. PLoS One. 2023;18(12):e0295418. 38064447 10.1371/journal.pone.0295418 PMC10707584

[24] Fernández‐de‐Las‐Peñas C , Cancela‐Cilleruelo I , Moro‐López‐Menchero P , et al. Exploring the trajectory curve of long‐term musculoskeletal post‐COVID pain symptoms in hospitalized COVID‐19 survivors: a multicenter study. Pain. 2023;164(2):413‐420. 35930390 10.1097/j.pain.0000000000002718

[25] Fernández‐de‐Las‐Peñas C , Torres‐Macho J , Guijarro C , Martín‐Guerrero JD , Pellicer‐Valero OJ , Plaza‐Manzano G . Trajectory of gastrointestinal symptoms in previously hospitalized COVID‐19 survivors: the long COVID experience multicenter study. Viruses. 2023;15(5):1134. 37243220 10.3390/v15051134 PMC10221203

[26] Huang C , Huang L , Wang Y , et al. 6‐month consequences of COVID‐19 in patients discharged from hospital: a cohort study. Lancet. 2023;401(10393):e21‐e33. 37321233 10.1016/S0140-6736(23)00810-3 PMC10258565

[27] Hastie CE , Lowe DJ , McAuley A , et al. True prevalence of long‐COVID in a nationwide, population cohort study. Nat Commun. 2023;14(1):7892. 38036541 10.1038/s41467-023-43661-w PMC10689486

[28] Davis HE , McCorkell L , Vogel JM , Topol EJ . Long COVID: major findings, mechanisms and recommendations. Nat Rev Microbiol. 2023;21(3):133‐146. 36639608 10.1038/s41579-022-00846-2 PMC9839201

[29] Lu L , Chen L , Wang P , et al. Neurological complications during the Omicron COVID‐19 wave in China: a cohort study. Eur J Neurol. 2024;31(1):e16096. 37877685 10.1111/ene.16096 PMC11235988

[30] Kandemir H , Bülbül GA , Kirtiş E , Güney S , Sanhal CY , Mendilcioğlu İİ . Evaluation of long‐COVID symptoms in women infected with SARS‐CoV‐2 during pregnancy. Int J Gynaecol Obstet. 2024;164(1):148‐156. 37387323 10.1002/ijgo.14972

[31] Atchison CJ , Davies B , Cooper E , et al. Long‐term health impacts of COVID‐19 among 242,712 adults in England. Nat Commun. 2023;14(1):6588. 37875536 10.1038/s41467-023-41879-2 PMC10598213

[32] Stephenson T , Pinto Pereira SM , Shafran R , et al. Physical and mental health 3 months after SARS‐CoV‐2 infection (long COVID) among adolescents in England (CLoCk): a national matched cohort study. Lancet Child Adolesc Health. 2022;6(4):230‐239. 35143770 10.1016/S2352-4642(22)00022-0 PMC8820961

[33] Bertollo AG , Leite Galvan AC , Dama Mingoti ME , Dallagnol C , Ignácio ZM . Impact of COVID‐19 on anxiety and depression—biopsychosocial factors. CNS Neurol Disord Drug Targets. 2024;23(1):122‐133. 36809942 10.2174/1871527322666230210100048

[34] Nalbandian A , Sehgal K , Gupta A , et al. Post‐acute COVID‐19 syndrome. Nat Med. 2021;27(4):601‐615. 33753937 10.1038/s41591-021-01283-z PMC8893149

[35] Ceban F , Ling S , Lui L , et al. Fatigue and cognitive impairment in post‐COVID‐19 syndrome: a systematic review and meta‐analysis. Brain Behav Immun. 2022;101:93‐135. 34973396 10.1016/j.bbi.2021.12.020 PMC8715665

[36] Latronico N , Peli E , Calza S , et al. Physical, cognitive and mental health outcomes in 1‐year survivors of COVID‐19‐associated ARDS. Thorax. 2022;77(3):300‐303. 34588274 10.1136/thoraxjnl-2021-218064

[37] Douaud G , Lee S , Alfaro‐Almagro F , et al. SARS‐CoV‐2 is associated with changes in brain structure in UK Biobank. Nature. 2022;604(7907):697‐707. 35255491 10.1038/s41586-022-04569-5 PMC9046077

[38] Yang AC , Kern F , Losada PM , et al. Dysregulation of brain and choroid plexus cell types in severe COVID‐19. Nature. 2021;595(7868):565‐571. 34153974 10.1038/s41586-021-03710-0 PMC8400927

[39] Alonso‐Lana S , Marquié M , Ruiz A , Boada M . Cognitive and neuropsychiatric manifestations of COVID‐19 and effects on elderly individuals with dementia. Front Aging Neurosci. 2020;12:588872. 33192483 10.3389/fnagi.2020.588872 PMC7649130

[40] Zhao Y , Li W , Lukiw W . Ubiquity of the SARS‐CoV‐2 receptor ACE2 and upregulation in limbic regions of Alzheimer's disease brain. Folia Neuropathol. 2021;59(3):232‐238. 34628788 10.5114/fn.2021.109495

[41] Pérez‐Rodríguez P , Díaz de Bustamante M , Aparicio Mollá S , et al. Functional, cognitive, and nutritional decline in 435 elderly nursing home residents after the first wave of the COVID‐19 pandemic. Eur Geriatr Med. 2021;12(6):1137‐1145. 34165775 10.1007/s41999-021-00524-1 PMC8222945

[42] Gu J , Liu Q , Zhang J , Xu S . COVID‐19 and trained immunity: the inflammatory burden of long covid. Front Immunol. 2023;14:1294959. 38090572 10.3389/fimmu.2023.1294959 PMC10713746

[43] Qiu Y , Mo C , Xu S , et al. Research progress on perioperative blood‐brain barrier damage and its potential mechanism. Front Cell Dev Biol. 2023;11:1174043. 37101615 10.3389/fcell.2023.1174043 PMC10124715

[44] von Bartheld CS , Wang L . Prevalence of olfactory dysfunction with the omicron variant of SARS‐CoV‐2: a systematic review and meta‐analysis. Cells. 2023;12(3):430. 36766771 10.3390/cells12030430 PMC9913864

[45] Chen S , Liang J , Chen D , et al. Cerebrospinal fluid metabolomic and proteomic characterization of neurologic post‐acute sequelae of SARS‐CoV‐2 infection. Brain Behav Immun. 2024;115:209‐222. 37858739 10.1016/j.bbi.2023.10.016

[46] Meinhardt J , Streit S , Dittmayer C , Manitius RV , Radbruch H , Heppner FL . The neurobiology of SARS‐CoV‐2 infection. Nat Rev Neurosci. 2024;25(1):30‐42. 38049610 10.1038/s41583-023-00769-8

[47] Xu E , Xie Y , Al‐Aly Z . Long‐term neurologic outcomes of COVID‐19. Nat Med. 2022;28(11):2406‐2415. 36138154 10.1038/s41591-022-02001-z PMC9671811

[48] Dondaine T , Ruthmann F , Vuotto F , et al. Long‐term cognitive impairments following COVID‐19: a possible impact of hypoxia. J Neurol. 2022;269(8):3982‐3989. 35325308 10.1007/s00415-022-11077-z PMC8944178

[49] Jahani M , Dokaneheifard S , Mansouri K . Hypoxia: a key feature of COVID‐19 launching activation of HIF‐1 and cytokine storm. J Inflamm (Lond). 2020;17:33. 33139969 10.1186/s12950-020-00263-3 PMC7594974

[50] Serebrovska ZO , Chong EY , Serebrovska TV , Tumanovska LV , Hypoxia XiL . HIF‐1α, and COVID‐19: from pathogenic factors to potential therapeutic targets. Acta Pharmacol Sin. 2020;41(12):1539‐1546. 33110240 10.1038/s41401-020-00554-8 PMC7588589

[51] Soung AL , Vanderheiden A , Nordvig AS , et al. COVID‐19 induces CNS cytokine expression and loss of hippocampal neurogenesis. Brain. 2022;145(12):4193‐4201. 36004663 10.1093/brain/awac270 PMC9452175

[52] Fernández‐Castañeda A , Lu P , Geraghty AC , et al. Mild respiratory COVID can cause multi‐lineage neural cell and myelin dysregulation. Cell. 2022;185(14):2452‐2468. 35768006 10.1016/j.cell.2022.06.008 PMC9189143

[53] Monje M , Iwasaki A . The neurobiology of long COVID. Neuron. 2022;110(21):3484‐3496. 36288726 10.1016/j.neuron.2022.10.006 PMC9537254

[54] Fontes‐Dantas FL , Fernandes GG , Gutman EG , et al. SARS‐CoV‐2 Spike protein induces TLR4‐mediated long‐term cognitive dysfunction recapitulating post‐COVID‐19 syndrome in mice. Cell Rep. 2023;42(3):112189. 36857178 10.1016/j.celrep.2023.112189 PMC9935273

[55] Venkataramani V , Winkler F . Cognitive deficits in long COVID‐19. N Engl J Med. 2022;387(19):1813‐1815. 36351274 10.1056/NEJMcibr2210069

[56] Yang J , Wang W , Chen Z , et al. A vaccine targeting the RBD of the S protein of SARS‐CoV‐2 induces protective immunity. Nature. 2020;586(7830):572‐577. 32726802 10.1038/s41586-020-2599-8

[57] He C , Yang J , Hong W , et al. A self‐assembled trimeric protein vaccine induces protective immunity against Omicron variant. Nat Commun. 2022;13(1):5459. 36115859 10.1038/s41467-022-33209-9 PMC9482656

[58] Regev‐Yochay G , Gonen T , Gilboa M , et al. Efficacy of a fourth dose of COVID‐19 mRNA vaccine against omicron. N Engl J Med. 2022;386(14):1377‐1380. 35297591 10.1056/NEJMc2202542 PMC9006792

[59] Wang H , Zhang Y , Huang B , et al. Development of an inactivated vaccine candidate, BBIBP‐CorV, with potent protection against SARS‐CoV‐2. Cell. 2020;182(3):713‐721. e9. 32778225 10.1016/j.cell.2020.06.008 PMC7275151

[60] Ranzani OT , Hitchings M , Dorion M , et al. Effectiveness of the CoronaVac vaccine in older adults during a gamma variant associated pandemic of covid‐19 in Brazil: test negative case‐control study. BMJ. 2021;374:n2015. 34417194 10.1136/bmj.n2015 PMC8377801

[61] Voysey M , Clemens S , Madhi SA , et al. Safety and efficacy of the ChAdOx1 nCoV‐19 vaccine (AZD1222) against SARS‐CoV‐2: an interim analysis of four randomised controlled trials in Brazil, South Africa, and the UK. Lancet. 2021;397(10269):99‐111. 33306989 10.1016/S0140-6736(20)32661-1 PMC7723445

[62] Sadoff J , Gray G , Vandebosch A , et al. Safety and efficacy of single‐dose Ad26.COV2.S vaccine against Covid‐19. N Engl J Med. 2021;384(23):2187‐2201. 33882225 10.1056/NEJMoa2101544 PMC8220996

[63] Chen Y , Yang W , Chen F , Cui L . COVID‐19 and cognitive impairment: neuroinvasive and blood‒brain barrier dysfunction. J Neuroinflammation. 2022;19(1):222. 36071466 10.1186/s12974-022-02579-8 PMC9450840

[64] Chiew CJ , Premikha M , Chong CY , et al. Effectiveness of primary series and booster vaccination against SARS‐CoV‐2 infection and hospitalisation among adolescents aged 12–17 years in Singapore: a national cohort study. Lancet Infect Dis. 2023;23(2):177‐182. 36182678 10.1016/S1473-3099(22)00573-4 PMC9519171

[65] Arsenault C , Lewis TP , Kapoor NR , et al. Health system quality and COVID‐19 vaccination: a cross‐sectional analysis in 14 countries. Lancet Glob Health. 2024;12(1):e156‐e165. 38096888 10.1016/S2214-109X(23)00490-4 PMC10716622

[66] Lazarus JV , Wyka K , White TM , et al. A survey of COVID‐19 vaccine acceptance across 23 countries in 2022. Nat Med. 2023;29(2):366‐375. 36624316 10.1038/s41591-022-02185-4

[67] Pairo‐Castineira E , Rawlik K , Bretherick AD , et al. GWAS and meta‐analysis identifies 49 genetic variants underlying critical COVID‐19. Nature. 2023;617(7962):764‐768. 37198478 10.1038/s41586-023-06034-3 PMC10208981

[68] Kumar A , Narayan RK , Prasoon P , et al. COVID‐19 vaccination may enhance hippocampal neurogenesis in adults. Brain Behav Immun. 2023;107:87‐89. 36202167 10.1016/j.bbi.2022.09.020 PMC9527215

[69] Daly M , Robinson E . Depression and anxiety during COVID‐19. Lancet. 2022;399(10324):518. 10.1016/S0140-6736(22)00187-8 PMC8813060 35123689

[70] Yung CF , Pang D , Kam KQ , et al. BNT162b2 vaccine protection against omicron and effect of previous infection variant and vaccination sequence among children and adolescents in Singapore: a population‐based cohort study. Lancet Child Adolesc Health. 2023;7(7):463‐470. 37201540 10.1016/S2352-4642(23)00101-3 PMC10185330

[71] Yasmin F , Najeeb H , Naeem U , et al. Adverse events following COVID‐19 mRNA vaccines: a systematic review of cardiovascular complication, thrombosis, and thrombocytopenia. Immun Inflamm Dis. 2023;11(3):e807. 36988252 10.1002/iid3.807 PMC10022421

[72] Stamm TA , Partheymüller J , Mosor E , et al. Determinants of COVID‐19 vaccine fatigue. Nat Med. 2023;29(5):1164‐1171. 36973410 10.1038/s41591-023-02282-y PMC10202806

[73] Rubin R . Updated COVID‐19 vaccine now available in US, recommended for everyone older than 6 months. JAMA. 2023;330(15):1420. 37721745 10.1001/jama.2023.19759

[74] Simadibrata DM , Lesmana E , Gunawan J , Quigley EM , Simadibrata M . A systematic review of gut microbiota profile in COVID‐19 patients and among those who have recovered from COVID‐19. J Dig Dis. 2023;24(4):244‐261. 37265376 10.1111/1751-2980.13195

[75] Zhou B , Pang X , Wu J , Liu T , Wang B , Cao H . Gut microbiota in COVID‐19: new insights from inside. Gut Microbes. 2023;15(1):2201157. 37078497 10.1080/19490976.2023.2201157 PMC10120564

[76] Álvarez‐Santacruz C , Tyrkalska SD , Candel S . The microbiota in long COVID. Int J Mol Sci. 2024;25(2):1330. 38279329 10.3390/ijms25021330 PMC10816132

[77] Saviano A , Brigida M , Petruzziello C , et al. Intestinal damage, inflammation and microbiota alteration during COVID‐19 infection. Biomedicines. 2023;11(4):1014. 37189632 10.3390/biomedicines11041014 PMC10135602

[78] Zhang F , Lau RI , Liu Q , Su Q , Chan F , Ng SC . Gut microbiota in COVID‐19: key microbial changes, potential mechanisms and clinical applications. Nat Rev Gastroenterol Hepatol. 2023;20(5):323‐337. 36271144 10.1038/s41575-022-00698-4 PMC9589856

[79] Sefik E , Qu R , Junqueira C , et al. Inflammasome activation in infected macrophages drives COVID‐19 pathology. Nature. 2022;606(7914):585‐593. 35483404 10.1038/s41586-022-04802-1 PMC9288243

[80] Synodinou KD , Nikolaki MD , Triantafyllou K , Kasti AN . Immunomodulatory effects of probiotics on COVID‐19 infection by targeting the gut‐lung axis microbial cross‐talk. Microorganisms. 2022;10(9):1764. 36144365 10.3390/microorganisms10091764 PMC9505869

[81] Li J , Jing Q , Li J , et al. Assessment of microbiota in the gut and upper respiratory tract associated with SARS‐CoV‐2 infection. Microbiome. 2023;11(1):38. 36869345 10.1186/s40168-022-01447-0 PMC9982190

[82] Zhang D , Weng S , Xia C , et al. Gastrointestinal symptoms of long COVID‐19 related to the ectopic colonization of specific bacteria that move between the upper and lower alimentary tract and alterations in serum metabolites. BMC Med. 2023;21(1):264. 37468867 10.1186/s12916-023-02972-x PMC10355065

[83] Mancabelli L , Taurino G , Ticinesi A , et al. Disentangling the interactions between nasopharyngeal and gut microbiome and their involvement in the modulation of COVID‐19 infection. Microbiol Spectr. 2023;11(5):e0219423. 37728335 10.1128/spectrum.02194-23 PMC10581039

[84] Bose T , Wasimuddin , Acharya V , et al. A cross‐sectional study on the nasopharyngeal microbiota of individuals with SARS‐CoV‐2 infection across three COVID‐19 waves in India. Front Microbiol. 2023;14:1238829. 37744900 10.3389/fmicb.2023.1238829 PMC10511876

[85] Candel S , Tyrkalska SD , Álvarez‐Santacruz C , Mulero V . The nasopharyngeal microbiome in COVID‐19. Emerg Microbes Infect. 2023;12(1):e2165970. 36606725 10.1080/22221751.2023.2165970 PMC9869994

[86] Sencio V , Machelart A , Robil C , et al. Alteration of the gut microbiota following SARS‐CoV‐2 infection correlates with disease severity in hamsters. Gut Microbes. 2022;14(1):2018900. 34965194 10.1080/19490976.2021.2018900 PMC8726722

[87] Zhou T , Wu J , Zeng Y , et al. SARS‐CoV‐2 triggered oxidative stress and abnormal energy metabolism in gut microbiota. MedComm. 2022;3(1):e112. 35281785 10.1002/mco2.112 PMC8906553

[88] Liu S , Zhao Y , Feng X , Xu H . SARS‐CoV‐2 infection threatening intestinal health: a review of potential mechanisms and treatment strategies. Crit Rev Food Sci Nutr. 2023;63(33):12578‐12596. 35894645 10.1080/10408398.2022.2103090

[89] Tursi A , Papa A . Intestinal microbiome modulation during coronavirus disease 2019: another chance to manage the disease. Gastroenterology. 2022;162(7):2134. 32946905 10.1053/j.gastro.2020.08.056 PMC7492138

[90] Zhang F , Wan Y , Zuo T , et al. Prolonged impairment of short‐chain fatty acid and l‐isoleucine biosynthesis in gut microbiome in patients with COVID‐19. Gastroenterology. 2022;162(2):548‐561. e4. 34687739 10.1053/j.gastro.2021.10.013 PMC8529231

[91] Li Z , Zhu G , Lei X , et al. Genetic support of the causal association between gut microbiome and COVID‐19: a bidirectional Mendelian randomization study. Front Immunol. 2023;14:1217615. 37483615 10.3389/fimmu.2023.1217615 PMC10360131

[92] Nagai M , Moriyama M , Ishii C , et al. High body temperature increases gut microbiota‐dependent host resistance to influenza A virus and SARS‐CoV‐2 infection. Nat Commun. 2023;14(1):3863. 37391427 10.1038/s41467-023-39569-0 PMC10313692

[93] Trøseid M , Holter JC , Holm K , et al. Gut microbiota composition during hospitalization is associated with 60‐day mortality after severe COVID‐19. Crit Care. 2023;27(1):69. 36814280 10.1186/s13054-023-04356-2 PMC9946863

[94] Mannan A , Hoque MN , Noyon SH , et al. SARS‐CoV‐2 infection alters the gut microbiome in diabetes patients: a cross‐sectional study from Bangladesh. J Med Virol. 2023;95(4):e28691. 36946508 10.1002/jmv.28691

[95] Ng HY , Leung WK , Cheung KS . Association between gut microbiota and SARS‐CoV‐2 infection and vaccine immunogenicity. Microorganisms. 2023;11(2):452. 36838417 10.3390/microorganisms11020452 PMC9961186

[96] Mendes de Almeida V , Engel DF , Ricci MF , et al. Gut microbiota from patients with COVID‐19 cause alterations in mice that resemble post‐COVID symptoms. Gut Microbes. 2023;15(2):2249146. 37668317 10.1080/19490976.2023.2249146 PMC10481883

[97] Brogna C , Viduto V , Fabrowski M , et al. The importance of the gut microbiome in the pathogenesis and transmission of SARS‐CoV‐2. Gut Microbes. 2023;15(1):2244718. 37559387 10.1080/19490976.2023.2244718 PMC10416738

[98] Li J , Ghosh TS , McCann R , et al. Robust cross‐cohort gut microbiome associations with COVID‐19 severity. Gut Microbes. 2023;15(1):2242615. 37550964 10.1080/19490976.2023.2242615 PMC10411309

[99] Ancona G , Alagna L , Alteri C , et al. Gut and airway microbiota dysbiosis and their role in COVID‐19 and long‐COVID. Front Immunol. 2023;14:1080043. 36969243 10.3389/fimmu.2023.1080043 PMC10030519

[100] Moreno‐Corona NC , López‐Ortega O , Pérez‐Martínez CA , et al. Dynamics of the microbiota and its relationship with post‐COVID‐19 syndrome. Int J Mol Sci. 2023;24(19):14822. 37834270 10.3390/ijms241914822 PMC10573029

[101] Xiang H , Liu QP . Alterations of the gut microbiota in coronavirus disease 2019 and its therapeutic potential. World J Gastroenterol. 2022;28(47):6689‐6701. 36620345 10.3748/wjg.v28.i47.6689 PMC9813939

[102] Zhang J , Deng J , Li J , et al. Changes of gut microbiota under different nutritional methods in elderly patients with severe COVID‐19 and their relationship with prognosis. Front Immunol. 2023;14:1260112. 37781374 10.3389/fimmu.2023.1260112 PMC10533997

[103] Seong H , Kim JH , Han YH , et al. Clinical implications of gut microbiota and cytokine responses in coronavirus disease prognosis. Front Immunol. 2023;14:1079277. 37051240 10.3389/fimmu.2023.1079277 PMC10083496

[104] Nagata N , Takeuchi T , Masuoka H , et al. Human gut microbiota and its metabolites impact immune responses in COVID‐19 and its complications. Gastroenterology. 2023;164(2):272‐288. 36155191 10.1053/j.gastro.2022.09.024 PMC9499989

[105] Song J , Wu Y , Yin X , Ma H , Zhang J . The causal links between gut microbiota and COVID‐19: a Mendelian randomization study. J Med Virol. 2023;95(5):e28784. 37219044 10.1002/jmv.28784

[106] Eleftheriotis G , Tsounis EP , Aggeletopoulou I , et al. Alterations in gut immunological barrier in SARS‐CoV‐2 infection and their prognostic potential. Front Immunol. 2023;14:1129190. 37006316 10.3389/fimmu.2023.1129190 PMC10050566

[107] Shang W , Zhang S , Qian H , et al. Association of gut microbiota with COVID‐19 susceptibility and severity: a two‐sample Mendelian randomization study. J Med Virol. 2023;95(4):e28734. 37185856 10.1002/jmv.28734

[108] Chen H , Ye B , Su W , et al. The causal role of gut microbiota in susceptibility and severity of COVID‐19: a bidirectional Mendelian randomization study. J Med Virol. 2023;95(7):e28880. 37409643 10.1002/jmv.28880

[109] Wang M , Zhang Y , Li C , Chang W , Zhang L . The relationship between gut microbiota and COVID‐19 progression: new insights into immunopathogenesis and treatment. Front Immunol. 2023;14:1180336. 37205106 10.3389/fimmu.2023.1180336 PMC10185909

[110] Martín Giménez VM , Modrego J , Gómez‐Garre D , Manucha W , de Las Heras N . Gut microbiota dysbiosis in COVID‐19: modulation and approaches for prevention and therapy. Int J Mol Sci. 2023;24(15):12249. 37569625 10.3390/ijms241512249 PMC10419057

[111] Hwang IC , Vasquez R , Song JH , Engstrand L , Valeriano VD , Kang DK . Alterations in the gut microbiome and its metabolites are associated with the immune response to mucosal immunization with Lactiplantibacillus plantarum‐displaying recombinant SARS‐CoV‐2 spike epitopes in mice. Front Cell Infect Microbiol. 2023;13:1242681. 37705931 10.3389/fcimb.2023.1242681 PMC10495993

[112] Liang Z , Wang N , Fan C , et al. Disturbance of adaptive immunity system was accompanied by a decrease in plasma short‐chain fatty acid for patients hospitalized during SARS‐CoV‐2 infection after COVID‐19 vaccination. J Inflamm Res. 2023;16:5261‐5272. 38026252 10.2147/JIR.S434860 PMC10656857

[113] Ahmadi Badi S , Tarashi S , Fateh A , Rohani P , Masotti A , Siadat SD . From the role of microbiota in gut‐lung axis to SARS‐CoV‐2 pathogenesis. Mediators Inflamm. 2021;2021:6611222. 33953641 10.1155/2021/6611222 PMC8059477

[114] Plummer AM , Matos YL , Lin HC , et al. Gut‐brain pathogenesis of post‐acute COVID‐19 neurocognitive symptoms. Front Neurosci. 2023;17:1232480. 37841680 10.3389/fnins.2023.1232480 PMC10568482

[115] Malan‐Müller S , Valles‐Colomer M , Palomo T , Leza JC . The gut‐microbiota‐brain axis in a Spanish population in the aftermath of the COVID‐19 pandemic: microbiota composition linked to anxiety, trauma, and depression profiles. Gut Microbes. 2023;15(1):2162306. 36651663 10.1080/19490976.2022.2162306 PMC9851210

[116] Xiong RG , Li J , Cheng J , et al. The role of gut microbiota in anxiety, depression, and other mental disorders as well as the protective effects of dietary components. Nutrients. 2023;15(14):3258. 37513676 10.3390/nu15143258 PMC10384867

[117] Fu Y , Guo Z , Wang Y , et al. Single‐nucleus RNA sequencing reveals the shared mechanisms inducing cognitive impairment between COVID‐19 and Alzheimer's disease. Front Immunol. 2022;13:967356. 36211330 10.3389/fimmu.2022.967356 PMC9538863

[118] Zhou Y , Xu J , Hou Y , et al. Network medicine links SARS‐CoV‐2/COVID‐19 infection to brain microvascular injury and neuroinflammation in dementia‐like cognitive impairment. Alzheimers Res Ther. 2021;13(1):110. 34108016 10.1186/s13195-021-00850-3 PMC8189279

[119] Tremblay ME , Madore C , Bordeleau M , Tian L , Verkhratsky A . Neuropathobiology of COVID‐19: the role for glia. Front Cell Neurosci. 2020;14:592214. 33304243 10.3389/fncel.2020.592214 PMC7693550

[120] Samudyata , Oliveira AO , Malwade S , et al. SARS‐CoV‐2 promotes microglial synapse elimination in human brain organoids. Mol Psychiatry. 2022;27(10):3939‐3950. 36198765 10.1038/s41380-022-01786-2 PMC9533278

[121] Poloni TE , Medici V , Moretti M , et al. COVID‐19‐related neuropathology and microglial activation in elderly with and without dementia. Brain Pathol. 2021;31(5):e12997. 34145669 10.1111/bpa.12997 PMC8412067

[122] Matschke J , Lütgehetmann M , Hagel C , et al. Neuropathology of patients with COVID‐19 in Germany: a post‐mortem case series. Lancet Neurol. 2020;19(11):919‐929. 33031735 10.1016/S1474-4422(20)30308-2 PMC7535629

[123] Alexopoulos H , Magira E , Bitzogli K , et al. Anti‐SARS‐CoV‐2 antibodies in the CSF, blood‐brain barrier dysfunction, and neurological outcome: studies in 8 stuporous and comatose patients. Neurol Neuroimmunol Neuroinflamm. 2020;7(6):e893. 32978291 10.1212/NXI.0000000000000893 PMC7577546

[124] Franke C , Ferse C , Kreye J , et al. High frequency of cerebrospinal fluid autoantibodies in COVID‐19 patients with neurological symptoms. Brain Behav Immun. 2021;93:415‐419. 33359380 10.1016/j.bbi.2020.12.022 PMC7834471

[125] Franke C , Boesl F , Goereci Y , et al. Association of cerebrospinal fluid brain‐binding autoantibodies with cognitive impairment in post‐COVID‐19 syndrome. Brain Behav Immun. 2023;109:139‐143. 36657623 10.1016/j.bbi.2023.01.006 PMC9841734

[126] Geanes ES , LeMaster C , Fraley ER , et al. Cross‐reactive antibodies elicited to conserved epitopes on SARS‐CoV‐2 spike protein after infection and vaccination. Sci Rep. 2022;12(1):6496. 35444221 10.1038/s41598-022-10230-y PMC9019795

[127] Gasmi A , Kumar Mujawdiya P , Noor S , Piscopo S , Résimont S , Menzel A . Increasing efficacy of covid‐19 vaccines by lifestyle interventions. Arch Razi Inst. 2022;77(5):1527‐1538. 37123146 10.22092/ARI.2021.356491.1854 PMC10133642

[128] Romani A , Sergi D , Zauli E , et al. Nutrients, herbal bioactive derivatives and commensal microbiota as tools to lower the risk of SARS‐CoV‐2 infection. Front Nutr. 2023;10:1152254. 37324739 10.3389/fnut.2023.1152254 PMC10267353

[129] Shen Y , Dong Y , Jiao J , Wang P , Chen M , Li J . BBIBP‐CorV vaccination against the SARS‐CoV‐2 virus affects the gut microbiome. Vaccines (Basel). 2023;11(5):942. 37243047 10.3390/vaccines11050942 PMC10223200

[130] Zhao S , Lok K , Sin ZY , et al. COVID‐19 mRNA vaccine‐mediated antibodies in human breast milk and their association with breast milk microbiota composition. NPJ Vaccines. 2023;8(1):151. 37798293 10.1038/s41541-023-00745-4 PMC10556030

[131] Leung J . Interaction between gut microbiota and COVID‐19 and its vaccines. World J Gastroenterol. 2022;28(40):5801‐5806. 36353201 10.3748/wjg.v28.i40.5801 PMC9639653

[132] Hong M , Lan T , Li Q , et al. A comprehensive perspective on the interaction between gut microbiota and COVID‐19 vaccines. Gut Microbes. 2023;15(1):2233146. 37431857 10.1080/19490976.2023.2233146 PMC10337507

[133] Han M , Huang Y , Gui H , et al. Dynamic changes in host immune system and gut microbiota are associated with the production of SARS‐CoV‐2 antibodies. Gut. 2023;72(10):1996‐1999. 36207022 10.1136/gutjnl-2022-327561 PMC10511961

[134] Tang B , Tang L , He W , et al. Correlation of gut microbiota and metabolic functions with the antibody response to the BBIBP‐CorV vaccine. Cell Rep Med. 2022;3(10):100752. 36228621 10.1016/j.xcrm.2022.100752 PMC9589008

[135] Ng SC , Peng Y , Zhang L , et al. Gut microbiota composition is associated with SARS‐CoV‐2 vaccine immunogenicity and adverse events. Gut. 2022;71(6):1106‐1116. 35140064 10.1136/gutjnl-2021-326563 PMC8844967

[136] Huang G , Mao Y , Zhang W , et al. Explore the changes of intestinal flora in patients with coronavirus disease 2019 based on bioinformatics. Front Cell Infect Microbiol. 2023;13:1265028. 37900316 10.3389/fcimb.2023.1265028 PMC10611479

[137] Ale EC , Binetti AG . Role of probiotics, prebiotics, and synbiotics in the elderly: insights into their applications. Front Microbiol. 2021;12:631254. 33584631 10.3389/fmicb.2021.631254 PMC7876055

[138] Vaezi M , Ravanshad S , Akbari Rad M , Zarrinfar H , Kabiri M . The effect of synbiotic adjunct therapy on clinical and paraclinical outcomes in hospitalized COVID‐19 patients: a randomized placebo‐controlled trial. J Med Virol. 2023;95(2):e28463. 36602047 10.1002/jmv.28463

[139] Reino‐Gelardo S , Palop‐Cervera M , Aparisi‐Valero N , et al. Effect of an immune‐boosting, antioxidant and anti‐inflammatory food supplement in hospitalized COVID‐19 patients: a prospective randomized pilot study. Nutrients. 2023;15(7):1736. 37049576 10.3390/nu15071736 PMC10096722

[140] Forsgård RA , Rode J , Lobenius‐Palmér K , et al. Limosilactobacillus reuteri DSM 17938 supplementation and SARS‐CoV‐2 specific antibody response in healthy adults: a randomized, triple‐blinded, placebo‐controlled trial. Gut Microbes. 2023;15(1):2229938. 37401761 10.1080/19490976.2023.2229938 PMC10321188

[141] Gutiérrez‐Castrellón P , Gandara‐Martí T , Abreu Y , Abreu AT , et al. Probiotic improves symptomatic and viral clearance in Covid19 outpatients: a randomized, quadruple‐blinded, placebo‐controlled trial. Gut Microbes. 2022;14(1):2018899. 35014600 10.1080/19490976.2021.2018899 PMC8757475

[142] Saviano A , Potenza A , Siciliano V , et al. COVID‐19 pneumonia and gut inflammation: the role of a mix of three probiotic strains in reducing inflammatory markers and need for oxygen support. J Clin Med. 2022;11(13):3758. 35807040 10.3390/jcm11133758 PMC9267834

[143] De Boeck I , Cauwenberghs E , Spacova I , et al. Randomized, double‐blind, placebo‐controlled trial of a throat spray with selected Lactobacilli in COVID‐19 outpatients. Microbiol Spectr. 2022;10(5):e0168222. 36154666 10.1128/spectrum.01682-22 PMC9604152

[144] Di Pierro F , Iqtadar S , Mumtaz SU , et al. Clinical effects of Streptococcus salivarius K12 in hospitalized COVID‐19 patients: results of a preliminary study. Microorganisms. 2022;10(10):1926. 36296202 10.3390/microorganisms10101926 PMC9609702

[145] Nalbandian A , Desai AD , Wan EY . Post‐COVID‐19 condition. Annu Rev Med. 2023;74:55‐64. 35914765 10.1146/annurev-med-043021-030635

[146] Hartung TJ , Neumann C , Bahmer T , et al. Fatigue and cognitive impairment after COVID‐19: a prospective multicentre study. EClinicalMedicine. 2022;53:101651. 36133318 10.1016/j.eclinm.2022.101651 PMC9482331

[147] Liu YH , Wang YR , Wang QH , et al. Post‐infection cognitive impairments in a cohort of elderly patients with COVID‐19. Mol Neurodegener. 2021;16(1):48. 34281568 10.1186/s13024-021-00469-w PMC8287105

[148] Liu YH , Chen Y , Wang QH , et al. One‐year trajectory of cognitive changes in older survivors of COVID‐19 in Wuhan, China: a longitudinal cohort study. JAMA Neurol. 2022;79(5):509‐517. 35258587 10.1001/jamaneurol.2022.0461 PMC8905512

[149] Xavier‐Santos D , Padilha M , Fabiano GA , et al. Evidences and perspectives of the use of probiotics, prebiotics, synbiotics, and postbiotics as adjuvants for prevention and treatment of COVID‐19: a bibliometric analysis and systematic review. Trends Food Sci Technol. 2022;120:174‐192. 35002079 10.1016/j.tifs.2021.12.033 PMC8720301

[150] Zhu J , Pitre T , Ching C , Zeraatkar D , Gruchy S . Safety and efficacy of probiotic supplements as adjunctive therapies in patients with COVID‐19: a systematic review and meta‐analysis. PLoS One. 2023;18(3):e0278356. 37000812 10.1371/journal.pone.0278356 PMC10065254

[151] Giannoni E , Baud D , Agri VD , Gibson GR , Reid G . Probiotics and COVID‐19. Lancet Gastroenterol Hepatol. 2020;5(8):720‐721. 10.1016/S2468-1253(20)30195-3 PMC7357984 32673603

[152] Kim CS , Cha L , Sim M , et al. Probiotic supplementation improves cognitive function and mood with changes in gut microbiota in community‐dwelling older adults: a randomized, double‐blind, placebo‐controlled, multicenter trial. J Gerontol A Biol Sci Med Sci. 2021;76(1):32‐40. 32300799 10.1093/gerona/glaa090 PMC7861012

[153] Rathi A , Jadhav SB , Shah N . A randomized controlled trial of the efficacy of systemic enzymes and probiotics in the resolution of post‐COVID fatigue. Medicines (Basel). 2021;8(9):47. 34564089 10.3390/medicines8090047 PMC8472462

[154] Mozota M , Castro I , Gómez‐Torres N , et al. Administration of Ligilactobacillus salivarius MP101 in an elderly nursing home during the COVID‐19 pandemic: immunological and nutritional impact. Foods. 2021;10(9):2149. 34574259 10.3390/foods10092149 PMC8470390

[155] Mozota M , Castro I , Gómez‐Torres N , et al. Administration of Ligilactobacillus salivarius CECT 30632 to elderly during the COVID‐19 pandemic: nasal and fecal metataxonomic analysis and fatty acid profiling. Front Microbiol. 2022;13:1052675. 36590434 10.3389/fmicb.2022.1052675 PMC9800801

[156] Catinean A , Sida A , Silvestru C , Balan GG . Ongoing treatment with a spore‐based probiotic containing five strains of bacillus improves outcomes of mild COVID‐19. Nutrients. 2023;15(3):488. 36771194 10.3390/nu15030488 PMC9920365

[157] Nobile V , Puoci F . Effect of a multi‐strain probiotic supplementation to manage stress during the COVID‐19 pandemic: a randomized, double‐blind, placebo‐controlled, cross‐over clinical trial. Neuropsychobiology. 2023;82(2):61‐71. 36634645 10.1159/000527956 PMC9843736

[158] Neris Almeida Viana S , do Reis Santos Pereira T , de Carvalho Alves J , et al. Benefits of probiotic use on COVID‐19: a systematic review and meta‐analysis. Crit Rev Food Sci Nutr. 2022:1‐13. 10.1080/10408398.2022.2128713 36178362

[159] Ray M , Manjunath A , Halami PM . Effect of probiotics as an immune modulator for the management of COVID‐19. Arch Microbiol. 2023;205(5):182. 37031431 10.1007/s00203-023-03504-0 PMC10098245

[160] Akram N , Saeed F , Afzaal M , et al. Gut microbiota and synbiotic foods: unveiling the relationship in COVID‐19 perspective. Food Sci Nutr. 2023;11(3):1166‐1177. 36911846 10.1002/fsn3.3162 PMC10002946

[161] Sohail A , Cheema HA , Mithani MS , et al. Probiotics for the prevention and treatment of COVID‐19: a rapid systematic review and meta‐analysis. Front Nutr. 2023;10:1274122. 37964926 10.3389/fnut.2023.1274122 PMC10641770

[162] Batista KS , de Albuquerque JG , Vasconcelos M , et al. Probiotics and prebiotics: potential prevention and therapeutic target for nutritional management of COVID‐19. Nutr Res Rev. 2023;36(2):181‐198. 34668465 10.1017/S0954422421000317 PMC8593414

[163] Tian Y , Ran H , Wen X , et al. Probiotics improve symptoms of patients with COVID‐19 through gut‐lung axis: a systematic review and meta‐analysis. Front Nutr. 2023;10:1179432. 37284648 10.3389/fnut.2023.1179432 PMC10239816

[164] Saxami G , Kerezoudi EN , Eliopoulos C , Arapoglou D , Kyriacou A . The gut‐organ axis within the human body: gut dysbiosis and the role of prebiotics. Life (Basel). 2023;13(10):2023. 37895405 10.3390/life13102023 PMC10608660

[165] Wischmeyer PE , Tang H , Ren Y , et al. Efficacy of probiotic treatment as post‐exposure prophylaxis for COVID‐19: a double‐blind, placebo‐controlled randomized trial. Clin Nutr. 2024;43(1):259‐267. 38103462 10.1016/j.clnu.2023.11.043

[166] Lau HC , Ng SC , Yu J . Targeting the gut microbiota in coronavirus disease 2019: hype or hope. Gastroenterology. 2022;162(1):9‐16. 34508775 10.1053/j.gastro.2021.09.009 PMC8425294

[167] Alenazy MF , Aljohar HI , Alruwaili AR , et al. Gut microbiota dynamics in relation to long‐COVID‐19 syndrome: role of probiotics to combat psychiatric complications. Metabolites. 2022;12(10):912. 36295814 10.3390/metabo12100912 PMC9611210

[168] Mardi A , Kamran A , Pourfarzi F , et al. Potential of macronutrients and probiotics to boost immunity in patients with SARS‐COV‐2: a narrative review. Front Nutr. 2023;10:1161894. 37312883 10.3389/fnut.2023.1161894 PMC10259402

[169] Önning G , Montelius C , Hillman M , Larsson N . Intake of Lactiplantibacillus plantarum HEAL9 improves cognition in moderately stressed subjects: a randomized controlled study. Nutrients. 2023;15(15):3466. 37571403 10.3390/nu15153466 PMC10421450

[170] Zhang T , Gao G , Kwok LY , Sun Z . Gut microbiome‐targeted therapies for Alzheimer's disease. Gut Microbes. 2023;15(2):2271613. 37934614 10.1080/19490976.2023.2271613 PMC10631445

[171] Kim CS , Jung S , Hwang GS , Shin DM . Gut microbiota indole‐3‐propionic acid mediates neuroprotective effect of probiotic consumption in healthy elderly: a randomized, double‐blind, placebo‐controlled, multicenter trial and in vitro study. Clin Nutr. 2023;42(6):1025‐1033. 37150125 10.1016/j.clnu.2023.04.001

[172] Laterza L , Putignani L , Settanni CR , et al. Ecology and machine learning‐based classification models of gut microbiota and inflammatory markers may evaluate the effects of probiotic supplementation in patients recently recovered from COVID‐19. Int J Mol Sci. 2023;24(7):6623. 37047594 10.3390/ijms24076623 PMC10094838

[173] Duindam HB , Kessels R , van den Borst B , Pickkers P , Abdo WF . Long‐term cognitive performance and its relation to anti‐inflammatory therapy in a cohort of survivors of severe COVID‐19. Brain Behav Immun Health. 2022;25:100513. 36159208 10.1016/j.bbih.2022.100513 PMC9482799

[174] Bartos A , Weinerova J , Diondet S , Vales K . Effect of human probiotics on memory, psychological and biological measures in elderly: a study protocol of bi‐center, double‐blind, randomized, placebo‐controlled clinical trial (CleverAge Biota). Front Aging Neurosci. 2022;14:996234. 36437993 10.3389/fnagi.2022.996234 PMC9686296

[175] Ferreiro AL , Choi J , Ryou J , et al. Gut microbiome composition may be an indicator of preclinical Alzheimer's disease. Sci Transl Med. 2023;15(700):eabo2984. 37315112 10.1126/scitranslmed.abo2984 PMC10680783

[176] Kiani L . Early microbiome changes in neurodegenerative disease. Nat Rev Neurol. 2023;19(8):458. 37402802 10.1038/s41582-023-00848-5

[177] Gonçalves J , Borges TJ , de Souza A . Microbiota and the response to vaccines against respiratory virus. Front Immunol. 2022;13:889945. 35603203 10.3389/fimmu.2022.889945 PMC9122122

[178] Lunken GR , Golding L , Schick A , Majdoubi A , Lavoie PM , Vallance BA . Gut microbiome and dietary fibre intake strongly associate with IgG function and maturation following SARS‐CoV‐2 mRNA vaccination. Gut. 2023;73(1):208‐210. 36549875 10.1136/gutjnl-2022-328556 PMC10715502

[179] Daddi L , Dorsett Y , Geng T , et al. Baseline gut microbiome signatures correlate with immunogenicity of SARS‐CoV‐2 mRNA vaccines. Int J Mol Sci. 2023;24(14):11703. 37511464 10.3390/ijms241411703 PMC10380288

[180] Peng Y , Zhang L , Mok C , et al. Baseline gut microbiota and metabolome predict durable immunogenicity to SARS‐CoV‐2 vaccines. Signal Transduct Target Ther. 2023;8(1):373. 37743379 10.1038/s41392-023-01629-8 PMC10518331

[181] Alexander JL , Mullish BH , Danckert NP , et al. The gut microbiota and metabolome are associated with diminished COVID‐19 vaccine‐induced antibody responses in immunosuppressed inflammatory bowel disease patients. EBioMedicine. 2023;88:104430. 36634565 10.1016/j.ebiom.2022.104430 PMC9831064

[182] Biancolella M , Colona VL , Luzzatto L , et al. COVID‐19 annual update: a narrative review. Hum Genomics. 2023;17(1):68. 37488607 10.1186/s40246-023-00515-2 PMC10367267

[183] Oh S , Seo H . Dietary intervention with functional foods modulating gut microbiota for improving the efficacy of COVID‐19 vaccines. Heliyon. 2023;9(5):e15668. 37124341 10.1016/j.heliyon.2023.e15668 PMC10121067

[184] Huang B , Wang J , Li L . Recent five‐year progress in the impact of gut microbiota on vaccination and possible mechanisms. Gut Pathog. 2023;15(1):27. 37308966 10.1186/s13099-023-00547-y PMC10258485

[185] Seong H , Choi BK , Han YH , et al. Gut microbiota as a potential key to modulating humoral immunogenicity of new platform COVID‐19 vaccines. Signal Transduct Target Ther. 2023;8(1):178. 37137906 10.1038/s41392-023-01445-0 PMC10154741

[186] Jordan A , Carding SR , Hall LJ . The early‐life gut microbiome and vaccine efficacy. Lancet Microbe. 2022;3(10):e787‐e794. 36088916 10.1016/S2666-5247(22)00185-9

[187] Zhu F , Huang S , Liu X , et al. Safety and efficacy of the intranasal spray SARS‐CoV‐2 vaccine dNS1‐RBD: a multicentre, randomised, double‐blind, placebo‐controlled, phase 3 trial. Lancet Respir Med. 2023;11(12):1075‐1088. 37979588 10.1016/S2213-2600(23)00349-1 PMC10682370

[188] Krasaewes K , Chaiwarith R , Chattipakorn N , Chattipakorn SC . Profiles of gut microbiota associated with clinical outcomes in patients with different stages of SARS‐CoV‐2 infection. Life Sci. 2023;332:122136. 37783267 10.1016/j.lfs.2023.122136

[189] Brīvība M , Silamiķele L , Birzniece L , et al. Gut microbiome composition and dynamics in hospitalized COVID‐19 patients and patients with post‐acute COVID‐19 syndrome. Int J Mol Sci. 2024;25(1):567. 38203738 10.3390/ijms25010567 PMC10779053

[190] Peters V , van de Steeg E , van Bilsen J , Meijerink M . Mechanisms and immunomodulatory properties of pre‐ and probiotics. Benef Microbes. 2019;10(3):225‐236. 30827150 10.3920/BM2018.0066

[191] Handajani YS , Turana Y , Yogiara Y , et al. Effects of Tempeh probiotics on elderly with cognitive impairment. Front Aging Neurosci. 2022;14:891773. 35813939 10.3389/fnagi.2022.891773 PMC9263263

[192] Sun Y , Wang K , Zhao W . Gut microbiota in perioperative neurocognitive disorders: current evidence and future directions. Front Immunol. 2023;14:1178691. 37215136 10.3389/fimmu.2023.1178691 PMC10192759

[193] Zhang T , Wu X , Liu B , Huang H , Zhou C , Liang P . The contribution of probiotics for the double‐edge effect of cefazolin on postoperative neurocognitive disorders by rebalancing the gut microbiota. Front Neurosci. 2023;17:1156453. 37179548 10.3389/fnins.2023.1156453 PMC10174111

[194] Yang X , Yu D , Xue L , Li H , Du J . Probiotics modulate the microbiota‐gut‐brain axis and improve memory deficits in aged SAMP8 mice. Acta Pharm Sin B. 2020;10(3):475‐487. 32140393 10.1016/j.apsb.2019.07.001 PMC7049608

[195] Naomi R , Embong H , Othman F , Ghazi HF , Maruthey N , Bahari H . Probiotics for Alzheimer's disease: a systematic review. Nutrients. 2021;14(1):20. 35010895 10.3390/nu14010020 PMC8746506

[196] Roman P , Estévez AF , Miras A , et al. A pilot randomized controlled trial to explore cognitive and emotional effects of probiotics in fibromyalgia. Sci Rep. 2018;8(1):10965. 30026567 10.1038/s41598-018-29388-5 PMC6053373

[197] Valdebenito‐Navarrete H , Fuentes‐Barrera V , Smith CT , et al. Can probiotics, particularly Limosilactobacillus fermentum UCO‐979C and Lacticaseibacillus rhamnosus UCO‐25A, be preventive alternatives against SARS‐CoV‐2. Biology (Basel). 2023;12(3):384. 36979076 10.3390/biology12030384 PMC10045641

[198] Su S , Zhao Y , Zeng N , et al. Epidemiology, clinical presentation, pathophysiology, and management of long COVID: an update. Mol Psychiatry. 2023;28(10):4056‐4069. 37491461 10.1038/s41380-023-02171-3

[199] Pomilio AB , Vitale AA , Lazarowski AJ . COVID‐19 and Alzheimer's disease: neuroinflammation, oxidative stress, ferroptosis, and mechanisms involved. Curr Med Chem. 2023;30(35):3993‐4031. 36200215 10.2174/0929867329666221003101548

[200] Fratta Pasini AM , Stranieri C , Girelli D , Busti F , Cominacini L . Is ferroptosis a key component of the process leading to multiorgan damage in COVID‐19. Antioxidants (Basel). 2021;10(11):1677. 34829548 10.3390/antiox10111677 PMC8615234

[201] Zhang R , Sun C , Chen X , et al. COVID‐19‐related brain injury: the potential role of ferroptosis. J Inflamm Res. 2022;15:2181‐2198. 35411172 10.2147/JIR.S353467 PMC8994634

[202] Jankauskas SS , Kansakar U , Sardu C , et al. COVID‐19 causes ferroptosis and oxidative stress in human endothelial cells. Antioxidants (Basel). 2023;12(2):326. 36829885 10.3390/antiox12020326 PMC9952002

[203] Peleman C , Van Coillie S , Ligthart S , et al. Ferroptosis and pyroptosis signatures in critical COVID‐19 patients. Cell Death Differ. 2023;30(9):2066‐2077. 37582864 10.1038/s41418-023-01204-2 PMC10482958

